# Iron(ii) carboxylates and simple carboxamides: an inexpensive and modular catalyst system for the synthesis of PLLA and PLLA-PCL block copolymers†

**DOI:** 10.1039/d3ra03112h

**Published:** 2023-06-07

**Authors:** Sarah Kirchhecker, Ngoc Nguyen, Stefan Reichert, Karola Lützow, Paul S. Eselem Bungu, Axel Jacobi von Wangelin, Sebastian Sandl, Axel T. Neffe

**Affiliations:** a Institute of Active Polymers, Helmholtz-Zentrum Hereon Kantstr. 55 14513 Teltow Germany axel.neffe@hereon.de; b Institute for Technical and Macromolecular Chemistry, University of Hamburg Bundesstrasse 55 20146 Hamburg Germany; c Department of Chemistry, University of Hamburg Martin-Luther-King-Platz 6 20146 Hamburg Germany

## Abstract

The combination of inexpensive Fe(ii) acetate with low molecular weight aliphatic carboxamides *in situ* generates an effective catalyst system for the ring opening polymerisation of lactones. PLLAs were produced in melt conditions with molar masses of up to 15 kg mol^−1^, narrow dispersity (*Đ* = 1.03), and without racemisation. The catalytic system was investigated in detail with regard to Fe(ii) source, and steric and electronic effects of the amide's substituents. Furthermore, the synthesis of PLLA-PCL block copolymers of very low randomness was achieved. This commercially available, inexpensive, modular, and user-friendly catalyst mixture may be suitable for polymers with biomedical applications.

## Abstract 2

The combination of inexpensive Fe(ii) acetate with low molecular weight aliphatic carboxamides *in situ* generates an effective catalyst system for the ring opening polymerisation of lactones.

## Graphical abstract

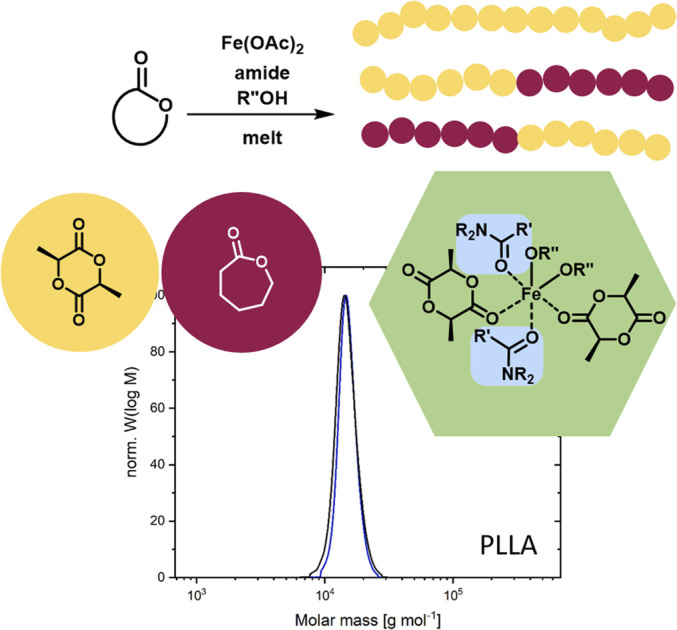

## Introduction

Polymers which can degrade over short and medium time spans are important in many different areas of application. Regenerative medicine^1,2^ requires biocompatible materials which can be resorbed by the body after a defined amount of time. Inbuilt degradability can also improve the environmental impact of single-use packaging,^3^ when recycling is not possible or not practised.^4,5^ Aliphatic polyesters, which can degrade by hydrolysis, are an important class of degradable materials. The major synthetic routes are polycondensation of diols and dicarboxylic acids and ring-opening (co)polymerisation (ROP) of lactones such as lactide, glycolide, ε-caprolactone or *para*-dioxanone.^6^ The latter method generally achieves higher molar masses and narrower dispersities.

Poly(l-lactic acid) (PLLA)^7,8^ and poly(ε-caprolactone) (PCL)^9,10^ are complimentary polymers with regard to their mechanical properties, drug permeabilities, and physiological degradation rates.^11,12^ Consequently, copolymers of the two monomers l-lactide and ε-caprolactone can provide materials with tailor-made properties for a wide range of applications. Precise control over the copolymerisation reaction enables the preparation of a range of different microstructures ranging from diblock to completely random distributions of the monomer units. Both starting materials and their copolymers are non-toxic and can be derived from biorenewable resources. Lactic acid (LA) for use in polymerisations can be produced by the fermentation of starch^13,14^ or non-edible lignocellulose^15^ and by chemical methods from cellulose^16^ or glycerol.^17,18^ Besides chemical manufacture, several syntheses of ε-caprolactone (CL) from biomass have been reported, such as *via* 5-hydroxymethylfurfural^19^ or enzymatic routes.^20^

ROP of lactones can be effected under various conditions with different catalysts, such as organocatalysts,^21,22^ enzymes,^23^ Lewis acids or bases,^24^ or metal–organic complexes.^25^ The latter class of catalysts, metal complexes, operate *via* a coordination-insertion mechanism and have been most widely employed.

Tin(ii) octanoate (Sn(Oct)_2_) is the prototypical catalyst with a well-established efficacy in the ROP of many lactones.^26,27^ Despite numerous investigations into alternative metal catalysts that provide good control of sequence structures^28,29^ and relative stereochemistries,^30^ tin(ii)octanoate remains the only commercially applied catalyst for the production of polylactide. Until very recently, it has not been surpassed under industrially relevant conditions.^31,32^ However, many tin compounds are toxic, severely limiting applications in biological settings such as the human body or ecosystems and requires careful removal of residuals.^33–36^ Tin(ii)octanoate, in particular, is suspected of damaging fertility or the unborn child and is harmful to aquatic life with long lasting effects such that the general use raises questions. The problematic eco-profile of tin-containing chemicals and chemical reactions has prompted great efforts toward the design of alternative metal catalysts for polyester synthesis, such as zinc^37–40^ and iron.

Iron is one of the most abundant metals on earth. As an essential nutrient and component of many human proteins, such as enzymes, its availability in the body is regulated *via* several transport and storage mechanisms. The high natural abundance of iron results in much lower market prices than tin, and its production rates are high and stable and have a low environmental impact.^41,42^ Still, comparatively little research has been directed at using inexpensive iron catalysts for effective ROP reactions.^43–51^ Several ligands have been evaluated in coordination complexes with Fe(ii) and Fe(iii) ions. Only a few catalysts were demonstrated to give reasonably good activity and control over the molar mass of the obtained polymer.^11,37,52^ Many of these studies were performed in solution, sometimes using the toxic propylene oxide as solvent and precursor to the initiating species.^53–57^ Polymerisation in the melt is preferable with regard to processing and environmental impact. The use of simple iron(iii) catalysts were reported for CL^58^ and LA,^59,60^ but in most cases, relatively high dispersities were obtained.^61,62^ Fe(ii) acetate (Fe(OAc)_2_) and several other Fe(ii) carboxylates were employed for the ROP of l-lactide ((l-LA)_2_) in the melt.^63^ They required high temperatures of 190–210 °C to proceed efficiently but could reach molar masses of up to 70 000 g mol^−1^ for Fe(OAc)_2_ and 150 000 g mol^−1^ for Fe(ii)isobutyrate. However, the dispersities were very broad, typically between 2.5 and 5. There was also evidence of racemisation at the required high reaction temperatures.

Recently, iron complexes with N–O donor ligands consisting of guanidines fused with benzoate esters were demonstrated to give faster rates than their zinc analogues and to catalyse lactide polymerisation at similar or even higher rates than Sn(Oct)_2_ under industrial conditions.^32,37^ The N–O ligand chelates the Fe *via* the N of guanidine and carbonyl O of the benzoate ester. Very recently, the same catalyst was successfully applied to the copolymerisation of LA and CL.^11^

**Scheme 1:**
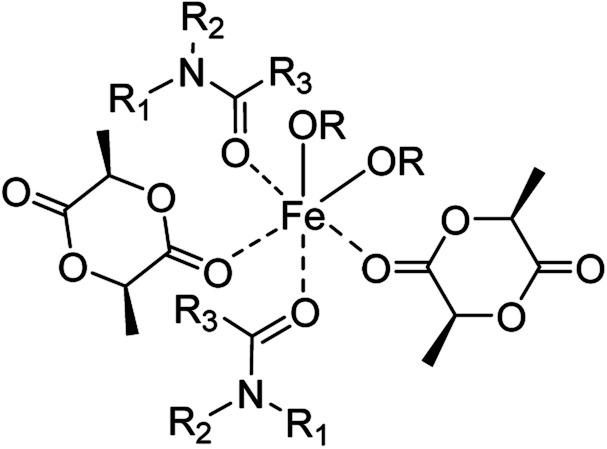
Illustration of a hypothetical iron(ii) complex bearing l-lactide, carboxamide, and alkoxide ligands.

It is important to note that in copolymerisation, the reaction rates of each monomer differ with different catalysts, and the rates directly impact the copolymer sequence structure. With large reactivity differences between the different monomers, fully random copolymers cannot be produced; rather, gradient structures are obtained. Sn(Oct)_2_ and other catalysts that allow rapid transesterifications between polyester chains can provide more randomized structures at long reaction times, but mostly with higher dispersities.^64^ For the synthesis of block copolymers, high transesterification rates are undesirable. Fully random copolymers of (l-LA)_2_ and CL are very difficult to prepare due to the significantly different reactivities with most catalysts. However, careful stereoelectronic ligand design can reduce LA polymerisation rates and produce nearly fully random copolymers.^65–67^ Catalysts of easily available, inexpensive, non-toxic metals that effectively catalyse the ROP of lactones under mild conditions and provide good control over molar mass, dispersity, and randomness are still in great demand. In the extension of the rather few literature reports on iron-catalysed ROP, our group has reported the successful polymerisation of morpholine-2,5-dione with Fe(OAc)_2_,^68^ and the beneficial effect of carboxamides as non-polymerisable catalytic adjuncts (NPCA) in the polymerisation of l-lactide by Fe(OAc)_2_.^69^ The addition of 10 wt% of the NPCA carboxamide (*vs.* the carbonyl units of the monomer) allowed for effective polymerisation at lower temperatures and with narrower dispersities than with Fe(OAc)_2_ alone. Our previous studies suggest that the carboxamide coordinates to the Fe centre *via* the carbonyl oxygen as a transient ligand (Scheme 1). In fact, the addition of *N*-methyl pyrrolidone (NMP), a cyclic tertiary amide, has been shown to enhance the catalytic activity of iron in cross-coupling reactions which enables the formation of catalytically active monomeric ferrates by coordination.^70–73^ The crucial role of the carboxamide was further investigated in this study by systematic variation of the amide substituents to assess the contribution of electronic and steric factors. In comparison, the effect of a defined Fe(ii) carboxylate consisting of a sterically demanding pivalate ligand, was investigated.^74^

The resulting polyesters were analysed by NMR, MALDI-ToF-MS, GPC, and polarimetry. The optimised catalytic system was then applied to the synthesis of PLLAs of different defined molar masses and the copolymerisation of (l-LA)_2_ and CL.

## Experimental

### Materials

l-Lactide and 4-*N*,*N*-dimethylaminoacetophenon (TCI, Eschborn, Germany), 1,8-octanediol (Sigma-Aldrich, Munich, Germany), *N-tert*-butylacetamide (Fluorochem, Hadfield, UK), and acetanilide (Merck, Darmstadt, Germany) were purified by recrystallisation (twice) from THF. 2-Chloro-*N*,*N*-diethylacetamide (Sigma-Aldrich, Munich, Germany), and 1,3-dimethyl-2-imidazolidone (ABCR, Karlsruhe, Germany) were dried over CaH_2_ at room temperature under stirring for 24 h, and after separation from the hydride degassed with nitrogen. *N*-Ethylacetamide, *N*,*N*-diethylacetamide, *N*,*N*-dimethylacetamide and tetramethylurea (Merck, Darmstadt, Germany) were dried over CaH_2_ at room temperature for 16 h, followed by distillation and storage under N_2_. Urea (Merck, Darmstadt, Germany or BASF, Ludwigshafen, Germany), was used as received or recrystallised from dry ethanol and stored under N_2_ until use. ε-Caprolactone (99%, Sigma-Aldrich, Munich, Germany) was distilled under vacuum and stored under N_2_ over molecular sieves. 1-Hexanol (Acros Organics, Nidderau, Germany; 99%, extra dry) was used as received. Iron(ii) acetate (99.995%, Sigma-Aldrich, Munich, Germany) was stored under N_2_ atmosphere.

### Methods

^1^H- and ^13^C-NMR spectra were recorded at 298 K on an Avance Neo 700 MHz spectrometer equipped with a triple resonance inverse Prodigy cryoprobe (Bruker, Rheinstetten, Germany; software Topspin version 4) in CDCl_3_. The determination of the number average molar mass *M*_*n*_ from the ^1^H spectra was performed by comparing the integrals of the CH_2_–O group of the initiator (4.11–3.96 ppm) with the CH protons of the lactic acid unit (5.33–4.86 and 4.37–4.22 ppm).

GPC measurements were carried out using chloroform as an eluent at 35 °C with a flow rate of 1 mL min^−1^ in the presence of 0.2 wt% toluene as internal standard. The 1260 Infinity II PSS Security GPC system (Polymer Standards Service, Mainz, Germany) was equipped with an isocratic pump, a degasser, an autosampler, a column-heating compartment, a UV and a RI detector. Two further detectors were used: a BI-MwA light scattering (Brookhaven Instruments, Holtsville, New York) and Security DVD 1260 viscometer (Polymer Standards Service GmbH, Mainz, Germany, PSS), which were used for universal calibration to determine absolute polymer molar mass. Size separation was achieved using a VS Lux, 10 μm, 50 mm × 8 mm ID pre-column and two SDV 10 μm, 300 mm × 8.0 mm ID analytical, linear XL columns (Polymer Standards Service GmbH, Mainz, Germany, PSS). Polymer molar masses were evaluated with the help of WINGPC UniChrom software Build 9050 (Polymer Standards Service GmbH, Mainz, Germany, PSS) using a universal calibration, which was obtained by applying polystyrene standards with *M*_*n*_ between 580 g mol^−1^ and 975 000 g mol^−1^ (Polymer Standards Service GmbH, Mainz, Germany).

To narrow down the molar mass heterogeneity of the PLLA-urea sample, preparative fractions were collected from the GPC eluent using the 1260 Infinity II fraction collector (Polymer Standards Service, Mainz, Germany). For the preparative fractionation experiment, a solution (8 wt%) was made by dissolving 160 mg in 20 mL chloroform. 100 μL of this solution was injected into the GPC column, and five fractions of 2 mL each were collected between 16.5 and 26.5 min elution times. The solvent was removed by evaporation, and the obtained fractions were dried overnight under a vacuum at room temperature. Sample recovery of approximately 90 wt% was achieved.

Differential scanning calorimetry (DSC) measurements were performed on a Netzsch DSC 204, Selb, Germany. The experiments were carried out under continuous nitrogen flow by heating a sample from room temperature up to 200 °C. This was followed by two cycles of cooling to −70 °C and heating to 200 °C at 10 K min^−1^.

The specific rotation [α]_D_ of polymer solutions in chloroform was measured at a concentration of 5 mg mL^−1^ in chloroform at 22 °C, using a P-2000 polarimeter (Jasco, Groß Umstadt, Germany).

Mass spectra were recorded on an UltrafleXtreme MALDI-ToF-MS spectrometer (Bruker, Bremen, Germany) equipped with a Smartbeam-II laser. *trans*-2-[3-(4-*tert*-Butylphenyl)-2-methyl-2-propenylidene]malononitrile (DCTB) was used as matrix and NaI or NaTFA to ionisation by Na^+^ addition. Polymer (5 mg mL^−1^ in THF or CHCl_3_), matrix (10 mg mL^−1^ in THF) and sodium ion source (10 mg mL^−1^ in THF) were mixed in a 1 : 10 : 1 or 5 : 10 : 1 volume ratio and then 1 μL pipetted on the matrix target for analysis. Some samples were also measured on a Bruker UltrafleXtreme but using DHB as matrix without an additional sodium source.

### Synthesis

#### Synthesis of iron(ii) pivalate

The synthesis of iron(ii) pivalate was performed according to a modified literature procedure.^75^ In a Schlenk flask equipped with condenser and relief valve, pivalic acid (11.2 mmol, 12.6 mL), pivalic acid anhydride (7 μmol, 1.2 mL) and iron powder (39.5 mmol, 2.2 g) were mixed under argon and heated to 170 °C for 15 h, at which time a highly viscous green solution was received. Pivalic acid was removed under reduced pressure at 100 °C, yielding a white/green solid that was washed with diethyl ether (2 × 30 mL). Excess solvent was removed under reduced pressure, and the raw product was dissolved in THF (40 mL) and filtered over Celite, finally adding 60 mL diethyl ether. 20 mL hexane was added, the ether was removed under reduced pressure, and a further 50 mL hexane was added. The product crystallised in green crystals, which were separated by filtration and dried under high vacuum. Yield: 0.94 g (64%)

Elementary analysis: found: C: 46.76%, H: 7.08%; calc. for C_10_H_18_FeO_4_: C: 46.54%, H: 7.03%.

#### Fe(OAc)_2_ catalysed ROP of l-lactide in the presence of carboxamides

In a typical experiment, 1.50 g (10.4 mmol) l-lactide, 14.6 mg (0.1 mmol, 1/104 equiv.) 1,8-octanediol, and 11.2 mg (0.065 mmol, 1/160 equiv.) (FeOAc)_2_ were mixed under N_2_ atmosphere. The respective carboxamide (2.08 mmol, 0.1 equiv.) was added, and the mixture was heated to 105 °C under stirring. After 3 h the reaction was stopped by removing it from the heat, and conversion was determined by ^1^H-NMR. The polymer was dissolved in 8–10 mL chloroform, precipitated in 500 mL cold methanol, washed again with cold methanol and dried under vacuum at 50 °C for 48 h.

^1^H-NMR (700 MHz, CDCl_3_) *δ* = 5.17–5.14 (q, *J* = 7.1 Hz, 200H, CH_α_), 4.37–4.33 (q, *J* = 6.7 Hz, 2H, CH_α_ next to initiator), 4.11 (tt, *J* = 10.8, 4.3 Hz, 4H, CH_2_O initiator), 1.86–1.47 (m, 647H, CH_3_ plus CH_2_ and CH_3_ of initiator) ppm;

^13^C-NMR (176 MHz, CDCl_3_) *δ* = 169.74, (CHCOO), 69.14, (OCHCO), 65.70 (OCH_2_CH_2_), 29.15, (OCH_2_*C*H_2_), 28.55, (2 CH_2_CH_2_CH_2_CH_2_O), 25.7,77 (2 CH_2_CH_2_CH_2_CH_2_O), 16.78 (CHCH_3_) ppm.

#### Fe(OAc)_2_ catalysed ROP of l-lactide with different target molar masses

The polymerisations were performed as above using DEAA (*N*,*N*-diethylacetamide) as the amide. 1,8-Octanediol was used as the initiator at an appropriate amount, ranging from 1/34 equiv. for a target molar mass of 5 kDa to 1/694 equiv. for a target molar mass of 100 kDa. The reactions were allowed to continue until all monomer was used up as assessed by ^1^H-NMR.

#### Fe(OAc)_2_ catalysed ROP of l-lactide and caprolactone

In a typical experiment for random copolymerisations, 0.505 g (3.5 mmol) l-lactide and 11.4 mg (0.066 mmol, 1/160 equiv.) Fe(OAc)_2_ were added to a dry Schlenk flask under N_2_. 0.78 mL (7 mmol) CL, 24.9 μL (0.198 mmol) 1-hexanol and 0.26 mL (2.1 mmol) of DEAA were added, and the flask was heated in an oil bath to the desired temperature (see results part; typically 105 °C or 140 °C) under magnetic stirring. Progress of the reaction was checked by ^1^H-NMR, and it was stopped when the monomers were fully consumed. For block copolymerisations, the procedure was the same, except that the second monomer was added to the reaction after full conversion of the first monomer was reached (by ^1^H-NMR check). The workup was performed in the same way as for pure PLLA.

^1^H-NMR (700 MHz, CDCl_3_) *δ* = 5.16 (q, *J* = 7.1 Hz, CH, LA–LA dyad, 40H), 5.11 (q, *J* = 7.2 Hz, LA-CL dyad; partially overlapping with LA–LA dyad, ∼2H), 4.35 (q, Ha next to initiator, 1H), 4.21–4.11 (m, O–CH_2_, CL–LA dyad, 4H, overlapping with CH_2_O of hexanol next to a LA (if existing)), 4.05 (t, *J* = 6.7 Hz, O–CH_2_, CL–CL dyad, 88H), 2.39 (t, *J* = 7.5 Hz, CO–CH_2_, LA–CL dyad, 2H), 2.30 (t, *J* = 7.5 Hz, CL–CL dyad, 88H), 1.66–1.61 (m, 182H), 1.59–1.56 (m, 118H), 1.50–1.46 (m, CH_2_ hexanol, 8H), 1.41–1.35 (m, 88H, CH_3_), 1.31–1.26 (CH_2_ hexanol, 8H), 0.88 (t, *J* = 6.8 Hz, 3H, CH_3_ of hexanol) ppm.

^13^C-NMR (176 MHz, CDCl_3_) *δ* = 173.67, 169.67, 69.13, 64.64, 64.27, 34.24, 31.54, 28.48, 25.65, 24.70, 22.65, 20.66, 16.77, 14.12 ppm.

#### Fe(OAc)_2_ catalysed ROP of l-lactide and glycolide

The copolymerisation was performed as above, except that 0.406 g glycolide (3.5 mmol) was used instead of CL.

^1^H-NMR (700 MHz, CDCl_3_) *δ* = 5.39–5.07 (m, 92H), 4.98–4.55 (m, 135H), 4.39–4.22 (m, 2H), 4.20–4.07 (m, 2H), 3.49 (bs, 2H), 3.28 (m, 1H), 2.67 (bs, 1H), 1.77–1.38 (m, 304H), 1.39–1.24 (m, 8H), 1.14 (t, *J* = 7.3 Hz, 2H), 0.89 (t, *J* = 7.0 Hz, 3H) ppm; ^13^C-NMR (176 MHz, CDCl_3_) *δ* = 169.61, 169.54, 169.47, 169.33, 166.52, 166.44, 166.41, 166.36, 77.21, 77.03, 76.84, 72.47, 69.33, 69.22, 69.12, 69.02, 68.96, 60.95, 60.88, 60.82, 60.76, 60.67, 16.75, 16.70, 16.65, 16.61, 15.83, 13.97 ppm.

## Results & discussion

### Optimisation of catalyst system

#### Screening of carboxamide adjuncts

We recently reported the observation of a carboxamide-enhanced iron-catalysed polymerisation of l-lactide using *N*-ethyl acetamide (EAA) as NPCA.^69^ The polymerisation proceeded in good yield at low temperature and gave PLLA of very low dispersity and without racemisation of the monomer. In that report, it was also shown that omittance of the NPCA would lead to lower conversion and higher required temperatures. Herein, we systematically evaluate the effect of the structure of a broader range of the carboxamide NPCAs toward their effect on the melt polymerisation of l-(LA)_2_ (Table 1). In particular, the electronic and steric effects of the substituents in the NPCAs were varied. For analysis of the differences in reaction rate, the polymerisations were stopped after 3 h, prior to full conversion of the monomer. Monomer conversions, initiator incorporations, molar masses, dispersities, and amount of transesterification of the polymers were determined from NMR and MALDI-ToF-MS spectra (Table 1. For details on the calculations, see Fig. S1 and S2 in the ESI†). The rest of the polymer, which did not contain the initiator was assumed to be initiated by residual water present in the reaction and is in agreement with the MALDI-ToF-MS spectra indicating free carboxylic acid end groups.

**Table 1:** NMR and MALDI-ToF-MS analysis of PLLAs obtained by iron-catalysis with the addition of different carboxamides^a^

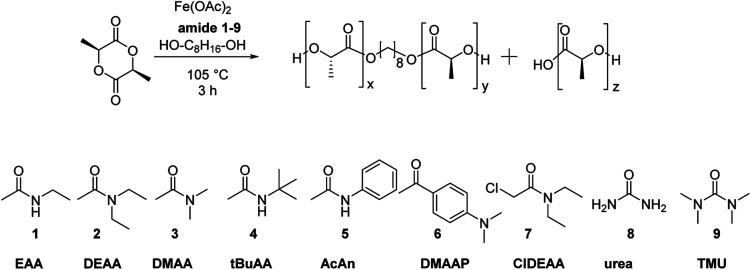							
Polymer	Conv. [%]	Ini % (NMR)	*M*_*n*_ NMR [g mol^−1^]	*M*_*n*_ MALDI [g mol^−1^]	*M*_w_ MALDI [g mol^−1^]	*Đ* MALDI	2*n*/(2*n* + 1) [%]
PLLA^69^	0	—	—	—	—	—	—
PLLA-EAA	77	74	8790	8655	9000	1.04	66
PLLA-DEAA	93	94	11 280	9780	10 120	1.03	79
PLLA-DMAA	93	99	9424	7745	7989	1.03	68
PLLA-*^t^*BuAA	44	75	4040	2395	3440	1.44	58
PLLA-AcAn	29	83	4160	2600	2990	1.15	67
PLLA-DMAAP	15	79	2440	1665	1825	1.10	69
PLLA-ClDEAA	39	89	4980	2670	3350	1.26	62
			4045	—	—	—	—
PLLA-urea^b^	88^c^	45	5490	6120	6255	1.02	78
			1572	2503	2652	1.06	—
PLLA-TMU	87	91	9885	9470	9820	1.04	68

a Reaction conditions: 105 °C, 3 h, 10 mol% carboxamide w.r.t. LA, initiator: 1/104 equiv. 1,8-octanediol (target molar mass: 15 kg mol^−1^).

b MALDI-ToF-MS values were determined separately for the octanediol- and water-initiated species using the fractionated samples.

c Some (l-LA)_2_ was lost from the reaction by sublimation to the neck of the flask. Conv.: conversion, ini %: percent of product molecules in which the initiator was incorporated, *M*_*n*_: number-average molar mass, *M*_w_: weight-average molar mass, *Đ*: dispersity, 2*n*/(2*n* + 1): relative content of polymers having 2*n* LA units, calculated from MALDI-ToF-MS spectra; polymers having 2*n* + 1 LA units could only be produced by transesterification.

**Fig. 1:**
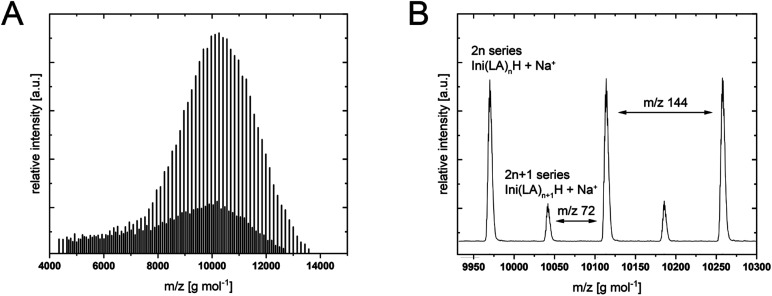
Representative MALDI-ToF-MS spectrum. (A) Full spectrum of PLLA-DEAA. (B) excerpt showing the two series (2*n* and (2*n* + 1)).

MALDI-ToF-MS analyses (Fig. 1 and ESI†) displayed two series, 2*n* and 2*n* + 1. The major polymer is the octanediol-initiated polymer (+Na^+^). The series of lower intensity has a mass difference of 72 g mol^−1^, which corresponds to one LA unit. The ROP of the lactide dimer leads to a polymer of even numbers of LA units (2*n*). Transesterification, however, can happen at any LA unit in the polymer chain, leading to the formation of PLLAs with uneven numbers of LA repeating units (2*n* + 1). The ratio of the intensities between these two series (2*n*/(2*n* + 1)) can therefore be used as a measure for the transesterification activity of the catalyst and the reaction conditions. The values are listed in Table 1, along with the number and weight average molar masses and dispersity (*Đ*) determined from the MALDI-ToF-MS spectra. In the lower molar mass range, some cyclics and methanol-terminated linear oligomers were detected, as well as H_2_O-initiated species, if these were long enough.

*N*-Ethyl acetamide (EAA, 1, entry 1)^69^ gave moderately high conversion (77%), initiator incorporation (74%), a low dispersity (1.04), and a molar polymer mass of ∼9000 g mol^−1^. Highest conversions (>90%), highest molar masses (>9000), lowest dispersities (1.03), and highest degree of initiator incorporations (>94%) were observed with the tertiary carboxamides DEAA (2), DMAA (3), and TMU (9), respectively (entries 2, 3, and 9). Employment of the bulky *tert*-butyl derivative (4), the anilide (5), the acetophenone derivative (6), or the chloro acetamide (7) afforded low conversions (15–44%), lower molar masses (<5000 g mol^−1^), and higher dispersities (>1.1–1.44). The unique stereoelectronic properties of urea (8) – as a primary amide derivative with a more basic oxygen atom donor – showed high monomer conversion. However, minor amounts of the (l-LA)_2_ monomer sublimed to the neck of the reaction flask, not participating in the further reaction. In the other polymerisations, this also occurred, but the refluxing of the other, in contrast to urea liquid NPCAs, washed back any sublimated (l-LA)_2_. Initiator incorporation was the lowest, with urea as NPCA (45%). The highest 2*n*/(2*n* + 1) ratio, and therefore the lowest transesterification activity was achieved with DEAA and urea (78–79%), while all other amides gave lower values (58–69%).

For the carboxamides 1–3 and 8-9, which showed promising results in the Fe-catalysed ROP, additional GPC and optical rotation measurements were performed (Table 2). Molar masses data determined by GPC are generally higher than those determined by MALDI-ToF-MS. Likely, the actual molar mass lies in between. However, the dispersity values from both measurements are in agreement. Carboxamides 1–3 and 9 with high initiator incorporation produced PLLAs with very low dispersities of 1.03–1.04. Additives 4–7 led to slightly higher dispersity values. The molar mass distribution for PLLA synthesised with urea 8 as NPCA was bimodal, still, when each peak was analysed separately by MALDI-ToF MS after fractionation by preparative GPC, the dispersities were very narrow (Table 1). In terms of molar masses, DEAA effected the highest polymer molar masses, followed by TMU, EAA and DMAA. The molar masses achieved with urea as NPCA were lower due to the bimodality and the sublimation of monomer during the reaction.

**Table 2:** GPC-derived molar masses and dispersities and optical rotation of some PLLA samples

Polymer	*M*_*n*_ (GPC) [g mol^−1^]	*M*_w_ (GPC) [g mol^−1^]	*Đ* (GPC)	[α]_D_^22^[°]
PLLA-EAA	14 100	14 700	1.04	−153
PLLA-DEAA	14 500	15 000	1.04	−154
PLLA-DMAA	12 200	13 000	1.06	−150
PLLA-urea^a^	3240 (bimodal)	5600	1.72	−147
PLLA-TMU	12 500	14 400	1.16	−151

a Some (l-LA)_2_ monomer was lost from the melt reaction by sublimation to the neck of the flask. *M*_*n*_: number-average molar mass, *M*_w_: weight-average molar mass, *Đ*: dispersity, [α]_D_^22^: specific optical rotation at 589 nm and 22 °C.

The specific optical rotation [α]_D_^22^ was measured to determine the degree of racemisation during the reaction. The specific optical rotation of commercial PLLA is −155 to −153° at 22 °C.^76,77^ Our previous report showed that by running the polymerisation at 105 °C, racemisation of (l-LA)_2_ could be prevented, but some racemisation was observed at 140 °C.^69^ This was confirmed by our measurements for amides 1–3 and 9. However, PLLA-urea showed a slightly lower value of −147°, which could indicate that some racemisation occurred, possibly due to urea's basic character.

Overall, the tertiary carboxamide DEAA was determined to be the most efficient NPCA of the tested series giving high molar masses, low dispersity, slightly lower initiator incorporation than the related DMAA, and significantly lower transesterification. The results with DMAA, a tertiary amide with shorter alkyl substituents, mainly differed in the amount of transesterification. These results indicate that the catalytic activity of iron is enhanced by the coordination of small, electron-donating carboxamides. Noteworthy, both DEAA and DMAA are used as solvents and are, therefore, inexpensive and widely available.

Further, the optimum amount of DEAA for the polymerisations was investigated. Reactions with 5 mol% of carboxamide (with regards to each LA carbonyl unit) only reached 50% conversion after 3 h. 10 and 20 mol% of DEAA gave very similar results in conversion and initiator incorporation. GPC analysis showed a slightly higher dispersity of 1.13 at increased NPCA loading. Increasing the amount of NPCA has no beneficial effect on the outcome of the reaction.

The addition of urea (8) to the Fe-catalysed ROP of LA produced polymers with a bimodal distribution (by GPC and MALDI-ToF-MS (Fig. S3.8A and B†)). NMR analysis indicated the formation of at least two polymer species (low initiator incorporation, 45%). The polymer sample was therefore fractionated by GPC, and the individual fractions were analysed by MALDI-ToF-MS (Fig. S3.8C in the ESI†). After fractionation, an end group analysis confirmed that the main polymer fraction is the octanediol-initiated PLLA with a molar mass *M*_*n*_ of 6120 g mol^−1^ and a dispersity *Đ* of 1.02. The second and smaller fraction was H_2_O-initiated PLLA. MALDI-ToF-MS analysis indicated a third minor polymer series (GPC showed a tiny third peak), which was not assigned. Purification of the urea by repeated recrystallisation from dry ethanol and drying under a vacuum did not improve the result. Further, sublimation of the LA monomer was observed.

#### Effect of Fe source

Finally, the effect of the Fe(ii) pre-catalyst on the polymerisation was investigated. Undissolved iron(ii) acetate is a polymeric network that contains various acetate binding modes and significant amounts of oxide and hydroxide functions.^78^

For comparison, Fe(ii) pivalate (Fe(OPiv)_2_), with a sterically demanding carboxylate and a defined polymeric structure, was freshly prepared and tested for (l-LA)_2_ polymerisation with the different carboxamides under the same conditions as above (Table S4.1†).^74^ The reaction rates with Fe(ii) pivalate were much faster, so the polymerisations were stopped after 1 h in order to compare the effects of the different NPCAs. Within the series of carboxamides, the trends for monomer conversion, initiator incorporation, and calculated molar masses of the polymers were very similar for Fe(OAc)_2_ and Fe(OPiv)_2_. Again DEAA gave the best results in terms of conversion and initiator incorporation. Interestingly, dimethylurea, which was used here instead of tetramethylurea, gave an even lower initiator incorporation than urea. Overall, the enhanced reaction rates observed with Fe(OPiv)_2_ support the hypothesised role of the amide to form monomeric iron complexes by coordination.

Freshly prepared Fe(OPiv)_2_ is sensitive to air and moisture and requires handling under inert conditions. Similar treatment should be followed when using freshly prepared Fe(OAc)_2_, while commercial samples of Fe(OAc)_2_ are more stable due to contamination with undefined amounts of hydrates, such as Fe(OAc)_2_·4H_2_O, hydroxides like Fe(OH)(OAc)_2_, Fe(OAc)_3_ and other impurities.^79,80^ It is therefore expected that an enhanced activity would be obtained with freshly prepared Fe(OAc)_2_ and dry reagents under rigorous exclusion of air and moisture.^63^

#### Evaluation of optimised catalyst for the synthesis of PLLA with different molar masses

Next, the ability of the Fe(OAc)_2_/DEAA catalyst system to produce PLLAs of different molar masses was investigated (Table 3). (l-LA)_2_ was polymerised with varying amounts of 1,8-octanediol as initiator, leaving the reaction to proceed until all monomer was consumed, as assessed by ^1^H-NMR. The reactions were performed at 105 °C except for the higher target molar masses of 50k and 100k, where the temperature had to be increased to 140 °C to shorten the reaction times. The amount of initiator incorporation decreased with increasing molar masses, from 99% for PLLA-DEAA-5k to 22% for PLLA-DEAA-100k, with residual water acting as the major initiator. This is common and also happens with other catalysts, such as Sn(Oct)_2_, as residual water cannot completely be excluded from the reaction mixture. The fastest conversion at 105 °C was observed for PLLA-15k, which was stopped after 8 h, while both PLLA-DEAA-5k and PLLA-DEAA-25k showed lower monomer conversion rates and were stopped after 22 h. The conversion of (l-LA)_2_ was above 90% at 140 °C after 3 h in the case of PLLA-DEAA-50k and PLLA-DEAA-100k, and the ROPs were continued to 22 h, at which time the full conversion was observed. GPC-derived molar masses were higher than those determined by MALDI-ToF-MS, with the calculated *M*_*n*_ from ^1^H-NMR spectra in between. The polymer dispersities *Đ* increased with decreasing amount of initiator due to the competing water-initiated polymerisation. Surprisingly, the dispersity for PLLA-DEAA-15k was quite high for the GPC result (1.81), possibly due to significant transesterification during the extended reaction times of 8 h. It became evident from this study that the Fe(OAc)_2_/DEAA catalyst appears to reach its limit under the used mixture and can produce PLLA with maximum molar masses of about 28 000 g mol^−1^ under these reaction conditions. The specific optical rotation showed slightly lower [α]_D_^22^ values for all polymerisations performed at longer reaction times, independent of the temperature. The largest value (−150°) indicates no or minor racemisation and was measured for PLLA-DEAA-15k, which was only reacted for 8 h.

**Table 3:** Analytical data for PLLA of different target masses^a^

Polymer	Target mass	Initiator [%]	Reaction time [h]	Temp [°C]	*M*_*n*_ (NMR) [g mol^−1^]	*M*_*n*_ (GPC) [g mol^−1^]	*M*_w_ (GPC) [g mol^−1^]	*Đ* (GPC)	*M*_*n*_ (MALDI) [g mol^−1^]	*M*_w_ (MALDI) [g mol^−1^]	*Đ* (MALDI)	2*n*: (2*n* + 1) [%]	[α]_D_^22^ [°]
PLLA-DEAA-5k	5k	99	22	105	3700	4700	4900	1.06	3800	3950	1.04	59	−136
PLLA-DEAA-15k	15k	90	8	105	12 400	15 000	18 500	1.81	9170	9650	1.05	60	−150
PLLA-DEAA-25k	25k	73	22	105	22 500	16 800	24 700	1.47	10 730	11 750	1.10	53	−148
PLLA-DEAA-50k	50k	45	22	140	24 400	12 700	27 700	2.18	—	—	—	—	−147
PLLA-DEAA-100k	100k	22	22	140	29 800	9200	27 900	3.03	—	—	—	—	−148

a All polymers were synthesised using 1/160 equiv. Fe(OAc)_2_, 10 mol% DEAA as the amide adjunct and an appropriate amount of 1,8-octanediol (ranging from 1/34 eq. to 1/694 equiv.) as initiator to reach the targeted mass. Temp: reaction temperature, *M*_*n*_ number-average molar mass, *M*_w_: weight-average molar mass, *Đ*: dispersity, 2*n*/(2*n* + 1): relative content of polymers having 2*n* LA units, calculated from MALDI-ToF-MS spectra, [α]_D_^22^: specific optical rotation at 589 nm and 22 °C.

### Copolymers

#### PLLA-PCL copolymers

**Scheme 2:**
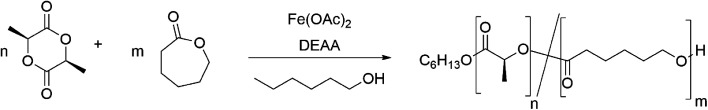
Copolymerisation of LA and CL using the Fe(OAc)_2_-amide catalyst system.

The optimised Fe(OAc)_2_/carboxamide catalyst system and conditions were now applied to the copolymerisation of caprolactone (CL) and lactide (l-(LA)_2_) (Scheme 2). The activity of the catalyst was evaluated in the individual homo-polymerisations of CL and LA, respectively, and of an equimolar mixture of both monomers under identical reaction conditions with 1-hexanol as initiator and a targeted molar mass of 10 000 g mol^−1^ (see S6 in the ESI†).

Lactide conversion was much slower with 1-hexanol than with octanediol as the initiator (Table 4). The target molar mass of 10k was achieved within 24 h and without racemisation of the lactide units. The dispersity was slightly higher than with octanediol (1.13 *vs.* 1.04). PCL-DEAA-hex had a much higher dispersity than PLLA-DEAA-hex (1.85, by GPC) and a lower molar mass (around half that of the target molar mass).

**Table 4:** Homopolymerisations of lactide and CL initiated with 1-hexanol

Polymer	Target mass	Initiator [%]	Reaction time [h]	Temp [°C]	*M*_*n*_ (NMR)	*M*_*n*_ (GPC)	*M*_w_ (GPC)	*Đ* (GPC)	*M*_*n*_ (MALDI)	*M*_w_ (MALDI)	*Đ* (MALDI)	2*n*/(2*n* + 1)	[α]_D_^22^
PLLA-DEAA-hex	10k	75	24	105	9866	9500	11 900	1.13	5400	5600	1.02	71%	−151
PCL-DEAA-hex	10k	n.d.^a^	19	140	3600^a^	4000	7400	1.85	2800	3200	1.13	—	—

a Initiator incorporation could not be calculated; *M*_*n*_ of hexanol-initiated PCL. Temp: reaction temperature, *M*_*n*_: number-average molar mass, *M*_w_: weight-average molar mass, *Đ*: dispersity, 2*n*/(2*n* + 1): relative content of polymers having 2*n* LA units, calculated from MALDI-ToF-MS spectra, [α]_D_^22^: specific optical rotation at 589 nm and 22 °C.

The catalyst activity was determined by the turnover number TON after 1 h reaction time: for (L-LA)_2_ : 90 (105 °C); for CL: 6 (105 °C), 61 (140 °C). It has been reported that the reaction rates of monomers can differ in the presence of other comonomers.^12,13,81^ Especially with catalysts based on Al, Mg and Zn, the rate of homopolymerisation of CL is often much faster than that of (l-LA)_2_.^82^ Here, this was not the case. The reaction rate of (l-LA)_2_ was much faster than that of CL in the homopolymerisations. The 1 : 1 mixture of both monomers showed the same conversion rate for l-(LA)_2_, but a lower rate for CL (87% after 24 h at 105 °C; nearly complete after 3 h at 140 °C). Copolymerisation at 140 °C is very likely to involve racemisation of lactic acid units. GPC, MALDI-ToF-MS and NMR analyses of the two homo-polymers are given in Table 4. The lactide homo-polymerisation took significantly longer with 1-hexanol than with octanediol, leading to lower incorporation of the initiator (75% *vs.* 93%). While the bifunctionality of the latter may play a role, the used 1-hexanediol may also have contained some water, which would simultaneously reduce the initiator incorporation and thus, the rate of reaction. The dispersity *Đ* of the hexanol-initiated polymer was higher than that derived from octanediol (1.13 *vs.* 1.04, by GPC). Optical rotations showed no significant racemisation in both cases. The caprolactone polymer PCL-DEAA-hex displayed much higher dispersity than the lactide polymer PLLA-DEAA-hex (1.85 *vs.* 1.13, by GPC) and a lower molar mass. It has been reported that enhanced transesterification takes place with less reactive monomers, as this reaction is energetically favoured (also see below).^11^ This may be the reason for the lower mass and higher dispersity found.

Copolymerisation reactions were performed with equimolar amounts of the two monomers under different reaction conditions and addition sequences to investigate the resulting copolymer architectures (Table 5). The target molar mass was kept at 10 000 g mol^−1^, and the reactions were allowed to proceed to completion. Where possible, the reaction temperature was kept at 105 °C to prevent racemisation of (l-LA)_2_, which led to very long reaction times for CL. Therefore, some experiments were also conducted at 140 °C. The copolymers were analysed by NMR and GPC (Table 5). The copolymer microstructure was analysed by ^1^H-NMR (see Fig. S.7†). The molar ratios of LA and CL incorporation were calculated from the integrals of the lactate methine resonance and the ε-CH_2_ resonance of CL in the polymers. The ratios of LA–CL hetero-dyads to homo-dyads (CL–CL, LA–LA) can be best calculated from the CL–CL and CL–LA dyads, as these are better separated.

**Table 5:** NMR and GPC analysis of PLLA-PCL copolymers^a^

Polymer	Reaction time	Temp [°C]	Conv LA [%]	Conv CL [%]	Molar ratio LA : CL	(LA–CL) rel. molar fraction	*l*_LA_	*l*_CL_	*R*	*M*_*n*_ (GPC)	*M*_w_ (GPC)	*Đ* (GPC)
PLLA-*grad*-PCL-105	7 d	105	>99	97	45 : 55	0.100	9.0	11	0.20	3300	6400	1.94
PLLA-*grad*-PCL-140	48 h	140	92	91	42 : 58	0.168	5.0	6.9	0.30	4000	12 900	3.22
PLLA-*b*-PCL-105	7 d	105	>99	91^b^	50 : 50	0.097	10.3	10.3	0.19	2500	7300	2.92
PCL-*b*-PLLA-140	48 h	140	86^c^	>99	27 : 73	0.028	19.3	52.1	0.07	5000	9100	1.82
PCL-*b*-PLLA-140-105	30 h	140/105	80^d^	97	47 : 53	0.021	44.3	50.2	0.04	7100	10 400	1.47

a The target molar mass was 10 000 g mol^−1^. The monomer feed was calculated to be equimolar in lactic acid and CL. The initiator was 1-hexanol (1/412 w.r.t. to LA + CL, Fe(OAc)_2_ was 1/160 equiv. of the cyclic monomers), amide: DEAA, 0.15 mol% (w.r.t. carbonyl units of the monomers). The reactions were allowed to continue until all (nearly all) monomer was used up, as assessed by ^1^H-NMR.

b CL was added after 4 h, when (l-LA)_2_ conv. was 99%.

c (l-LA)_2_ was added after 24 h. Some (l-LA)_2_ sublimed to the neck of flask and was lost from the reaction.

d (l-LA)_2_ was added after 4 h and temperature decreased to 105 °C. Temp: reaction temperature, Conv.: conversion of monomer, calculated from NMR, l: average block length of respective monomer; *R*: randomness; *M*_*n*_: number-average molar mass, *M*_w_: weight-average molar mass, *Đ*: dispersity, 2*n*/(2*n* + 1): relative content of polymers having 2*n* LA units, calculated from MALDI-ToF-MS spectra, [α]_D_^22^: specific optical rotation at 589 nm and 22 °C.

From these values the number average block lengths of each monomer can be calculated:^83,84^1
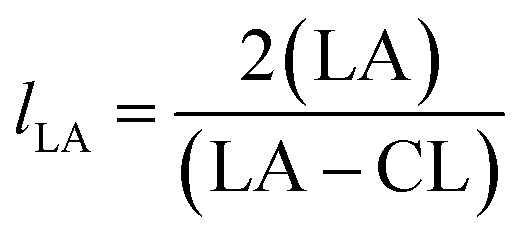
2
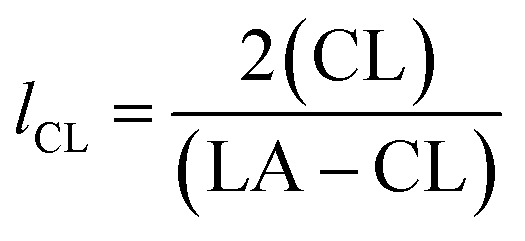
where (LA) and (CL) are the molar fractions of each respective monomer in the polymer and (LA–CL) the relative molar fraction of the hetero-dyads.

The randomness *R* of a polymer can be calculated from:3
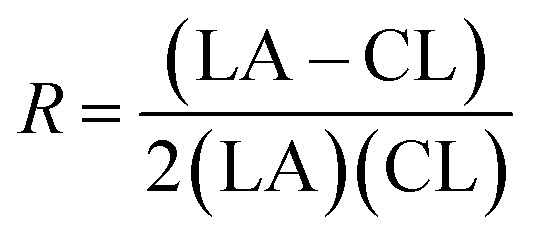


For a completely random polymer, *R* approaches 1, with values above 0.8 indicating significant randomness. Pure block copolymers have *R* = 0.

Random copolymerisations were attempted at 105 and 140 °C (entries 1 and 2, Table 5). A reaction time of 7 days was required for full conversion at 105 °C. The incorporation of both monomers was very similar and slightly lower for the lactide. The randomness was found to be low, which may indicate gradient structures in both cases due to the much higher reactivity of the lactide. The *R-*value of PLLA-ran-PCL-140 and the dispersity were higher than those of PLLA-ran-PCL-105, which supports the notion of active transesterification at higher temperatures. Alternative catalysts, such as Sn(Oct)_2_, produce much higher randomized structures than the Fe(OAc)_2_/DEAA catalyst *via* transesterification.^64^

Catalysts, which can only start block copolymerisations with one of the monomers, have been reported.^85,86^ We, therefore, performed reactions starting from (l-LA)_2_ (entry 3) and CL (entries 4 and 5). Incorporation of both monomers into the copolymer could be achieved *via* sequential addition starting with either monomer. However, due to the slow reaction rate of CL at 105 °C, synthesis of PLLA-*b*-PCL-105 took seven days. The randomness of PLLA-*b*-PCL-105 was similar to that of PLLA-*grad*-PCL-105 due to enhanced transesterification. At extended reaction times, transesterification becomes competitive with the slow CL incorporation, as also observed in a recent study with iron-guanidine catalysts.^11^PCL-*b*-PLLA block co-polymers with very low *R* values are given in entries 4 and 5. For PCL-*b*-PLLA-140-105, the temperature was lowered to 105 °C when the lactide was added after 4 h. Incorporation of lactide into PCL-*b*-PLLA-140 was lower as some of the (l-LA)_2_ sublimed from the reaction mixture. In all cases, the *R* values of PCL-*b*-PLLA are much lower than those of PLLA-*b*-PCL, possibly due to an initial faster incorporation of CL and lower transesterification.

The target molar mass for all reactions in Table 5 was 10 000 g mol^−1^, however, this mass was mostly not reached. The lowest mass was obtained from PLLA-*grad*-PCL-105, and the highest polymer masses from PCL-*b*-PLLA-140-105 (*M*_*n*_ = 7100 g mol^−1^, *M*_w_ = 10 400 g mol^−1^, by GPC). The latter stepwise procedure also afforded the far lowest dispersity of the co-polymers (*Đ* = 1.47).

DSC analysis was performed on some selected examples of copolymers. The results are summarised in Table S11.01.† Both the PLLA (163–168 °C) and PCL (33–51 °C) related melting transitions are relatively low, which indicates a small crystallite size, while the melting enthalpy indicated a low degree of crystallinity. In fact, in PLLA-*b*-PCL, no crystallisation of the PLLA was observed.

#### Transesterification

**Fig. 2:**
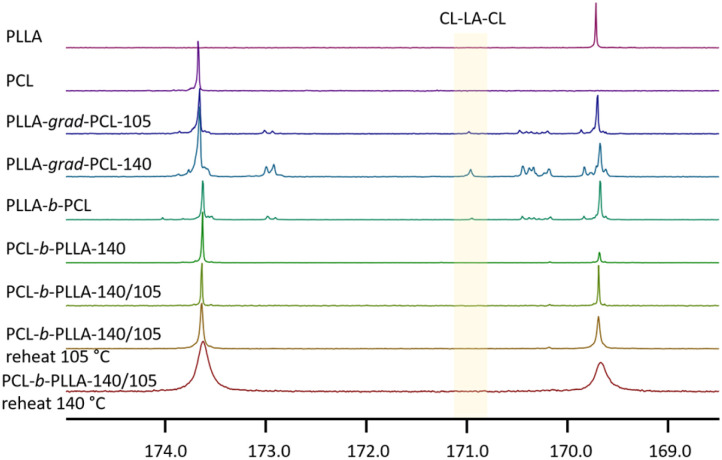
^13^C-NMR carbonyl regions of the l-lactide and CL homopolymers, copolymers, and block copolymers (in ppm).

This study of Fe(OAc)_2_/carboxamide-catalysed l-lactide polymerisations has documented only minor transesterification activity at 105 °C. Significant transesterification was however observed at long reaction times and elevated temperatures in the copolymerisation of (l-LA)_2_ and CL, however, even after 7 days at 105 °C no statistical copolymer was formed. A higher *R* value for PLLA-*b*-PCL compared to PCL-*b*-PLLA indicated that transesterification took place in that reaction. The degree of transesterification was determined from the carbonyl region of the ^13^C-NMR spectra.^87^ The CL–LA–CL triad containing only one lactic acid unit from the original lactide is produced by transesterification and gives a characteristic C=O resonance between 170.8 ppm (ref. 11) and 171.1 ppm.^67^ We observed the carbonyl signal of CL–LA–CL at 171.0 ppm in the spectra of the statistical copolymer and PLLA-*b*-PCL which confirmed that transesterification took place (Fig. 2). The signal is not present in the co-polymer that was initiated with the CL monomer.

Further, we studied potential transesterification reactions on the pre-formed copolymers under standard conditions. A mixture of PCL-*b*-PLLA-140-105, the copolymer displaying the lowest randomness value *R*, the catalyst Fe(OAc)_2_ and the carboxamide additive DEAA were heated for more than 2 days at 105 °C and 140 °C, respectively. No differences were detected in the ^13^C-NMR spectra before and after reaction. Our observations that transesterification was only observed in the block copolymers starting with lactide incorporation but was absent in the CL-initiated copolymers and when treating the final polymers under standard reaction conditions may be indicative of an active transesterification pathway during the slow CL insertion steps. The absence of transesterification in the addition sequence CL–(l-LA)_2_ makes the Fe(OAc)_2_/DEAA catalyst system ideally suited for the synthesis of PCL-PLLA block copolymers.

The synthesis of highly blocky copolymers in the melt is not an easy task, Sn(Oct)_2_ under similar conditions produces copolymers of much higher randomness.^88^ In our case, the sequential addition of monomer can produce real block copolymers. Where necessary, the polymerisation sequence can also be started with (l-LA)_2_, followed by CL at 140 °C, which should shorten the reaction times.

#### Glycolide copolymer

The copolymerisation of lactide with the hydroxyacetate dimer glycolide (GL) was investigated under similar conditions in the presence of the Fe(OAc)_2_/DEAA catalyst. The resulting PLLGA copolymers, poly[(l-lactic)-*co*-(glycolic acid)], find wide applications in biomedical settings.^89,90^ The homo-polymerisation of GL under the standard conditions solidified within 10 min at 105 °C to a polymer that could not be dissolved for further analysis. The equimolar copolymerisation of (l-LA)_2_ and GL under the same conditions as above proceeded much faster than the lactide polymerisation with this catalyst and afforded a highly crystalline copolymer that was insoluble in chloroform (the solvent of choice for GPC analyses). The PLLGA could be dissolved in DMSO, but GPC data from different solvents are difficult to compare. Structural analysis was performed by NMR spectroscopy in DMSO-d_6_ (Table S10.1†). The characteristic ^1^H-NMR signals largely overlapped. Therefore, the carbonyl resonances in the ^13^C-NMR spectra were used to calculate the ratio of dyads.^91^ The randomness for this copolymer was determined to be 0.35, which is higher than the highest value for the PLLA-PCL copolymers, but still very far from a real random distribution due to the large difference in monomer reactivities.

## Conclusions

In conclusion, a simple and inexpensive catalyst system was generated *in situ* from Fe(ii) acetate and 10 mol% of small aliphatic tertiary carboxamides. The catalyst is effective for the ROP of l-lactide, achieving PLLAs with very narrow dispersities (*Đ* = 1.03) up to a molar mass of 15 000 g mol^−1^ under melt conditions. When the polymerisation is performed at 105 °C and the reaction time is kept short enough, no racemisation of l-lactide occurs. Carboxamides with two small aliphatic substituents having a +I-effect seem to be most active in supporting the ROP by Fe(ii) carboxylates.

Furthermore, the optimised catalyst system was applied to the synthesis of PLLA-PCL copolymers, where it was found to be very useful for the synthesis of block copolymers of very low randomness due to its low transesterification activity, and the difference in reactivities of the two monomers. Starting from PCL, the catalyst can produce highly blocky copolymers with moderate dispersity at a target molar mass of 10 kg mol^−1^. The block-copolymerisations could be performed in the melt and without intermediate purification steps. This represents an inexpensive and simple way of synthesising PLLAs and block copolymers for use in applications such as the biomedical field and may have a lower environmental impact than the use of tin catalysts.

## Conflicts of interest

The authors declare no conflict of interest.

## Supplementary Material

RA-013-D3RA03112H-s001
