# Supplementary material for: Iron(ii) carboxylates and simple carboxamides: an inexpensive and modular catalyst system for the synthesis of PLLA and PLLA-PCL block copolymers
Source: RSC Adv. 2023 Jun 7;13(25):17102–13. doi: 10.1039/d3ra03112h (PMC10244980; doi:10.1039/d3ra03112h)
Supplement: RA-013-D3RA03112H-s001 [file RA-013-D3RA03112H-s001.pdf]

Electronic Supplementary Material (ESI) for RSC Advances.
This journal is © The Royal Society of Chemistry 2023

Iron(II) Carboxylates and Simple Carboxamides: An Inexpensive and Modular Catalyst System for the Synthesis of PLLA and PLLA-PCL Block Copolymers

Supporting Information

Sarah Kirchhecker[1], Ngoc Nguyen[2], Stefan Reichert[3], Karola Lützow[1], Paul Eselem Bungu[1], Axel Jacobi von Wangelin[3], Sebastian Sandl[3] and Axel T. Neffe[1,2*]

1: Institute for Active Polymers, Helmholtz Zentrum Hereon, Kantstr. 55, 14513 Teltow, Germany

2: Institute for Technical and Macromolecular Chemistry, University of Hamburg, Bundesstrasse 55, 20146 Hamburg, Germany

3: Department of Chemistry, University of Hamburg, Martin-Luther-King-Platz 6, 20146 Hamburg, Germany

*: to whom correspondence should be addressed: axel.neffe@hereon.de

S1. NMR spectrum of PLLA indicating peaks used for calculations

S2. Formulas used for calculations

S3. MALDI and GPC spectra of PLLA synthesized with different amide adjuncts

S4. PLLA polymerized with $Fe(OPv)_2$ and different amides as catalyst

S5. MALDI and GPC spectra of PLLA at different molar masses

S6. Kinetics of LL and CL homo- and copolymerisations

S7. NMR spectrum of copolymer indicating the peaks used for calculations

S8. GPC traces of copolymers

S9. MALDI spectra of selected copolymers

S10. Glycolide copolymerisation

S11. DSC investigations /thermal transitions

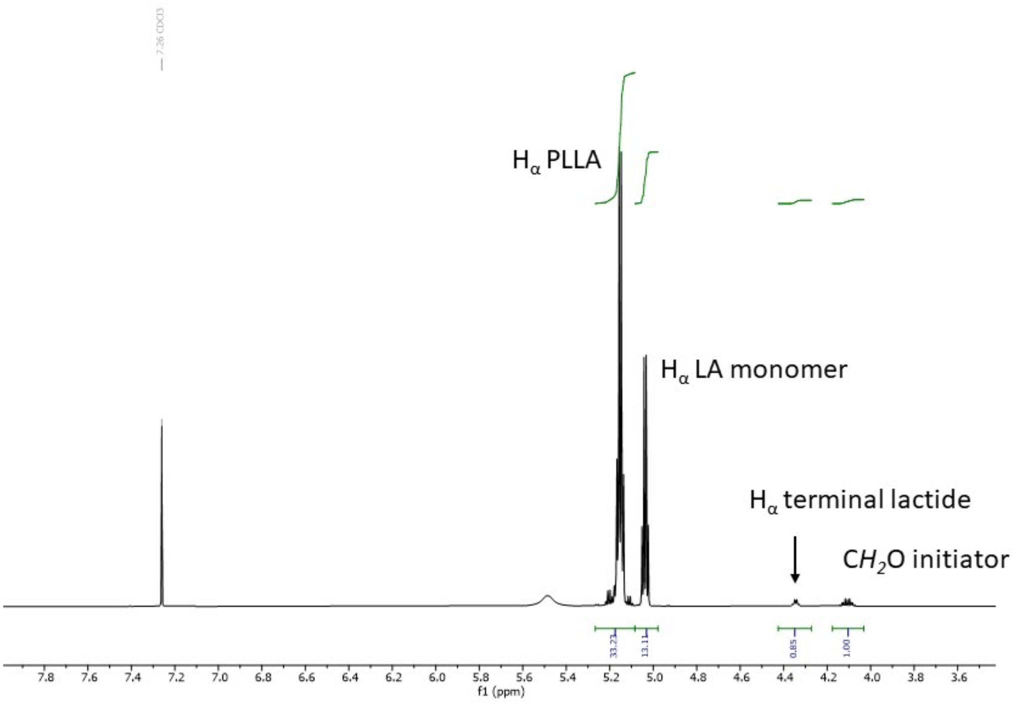

Figure S1. NMR spectrum of PLLA indicating the peaks used for calculation of conversion, M_n and incorporation of initiator

The determination of the number average molecular weight Mn from the ^{1}H spectra was performed by comparing the integrals of the CH_2-O group of the initiator (4.11–3.96 ppm) with the CH protons of the lactic acid unit (5.33–4.86 and 4.37–4.22 ppm).

S2. Formulas used for calculations in Table 1

Conversion = $$100 * \frac{(I_{H_{\alpha,polylactide}} + I_{H_{\alpha,terminal\ lactide}})}{(I_{H_{\alpha,polylactide}} + I_{H_{\alpha,terminal\ lactide}} + I_{H_{\alpha,monomer}})}$$ Eq. 1

%telechelic $$= 100\% \cdot \frac{I_{H_{\alpha,\ terminal\ lactide}}}{(I_{H_{\alpha,\ terminal\ lactide}} - \frac{I_{CH_2O_{Ini.}}}{2})}$$ Eq. 2

$M_{n,telechel}$ = $$2 \cdot \frac{2(I_{H_{\alpha,polylactide}} + I_{H_{\alpha,terminal\ lactide}})}{I_{CH_2O_{Ini.}}} \cdot 72.07\ g \cdot mol^{-1} + 146.227\ g \cdot mol^{-1}$$ Eq. 3

$$M_{n,OH\text{-}initialized} = (\frac{I_{H_{\alpha,polylactide}}}{H_{\alpha,terminal\ lactide}} + 1) \cdot 72.07\ g \cdot mol^{-1} + 18.015\ g \cdot mol^{-1} \quad \text{Eq. 4}$$

$$M_n(NMR) = \frac{\%\ telechelic}{100} \cdot M_{n,telechel} + (1 - \frac{\%\ telechelic}{100}) \cdot M_{n,OH\text{-}initialized} \quad \text{Eq. 5}$$

S3. MALDI spectra and GPC chromatogrammes of PLLA synthesized with different amides

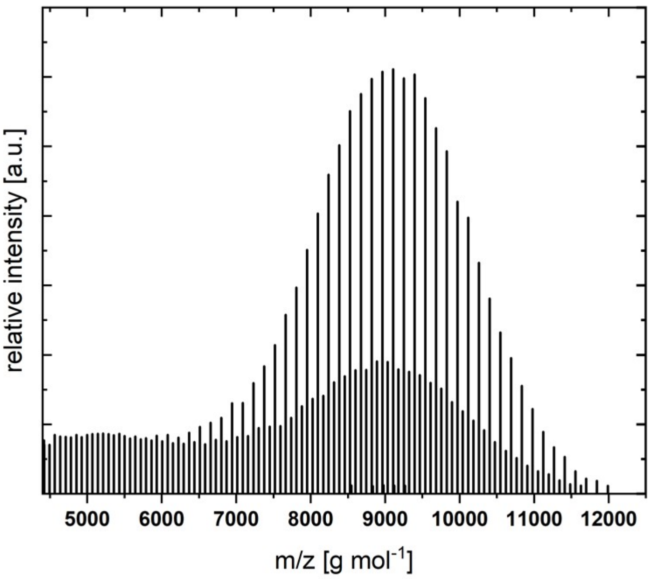

Figure S3.1.1 MALDI spectrum of **PLLA-EAA**

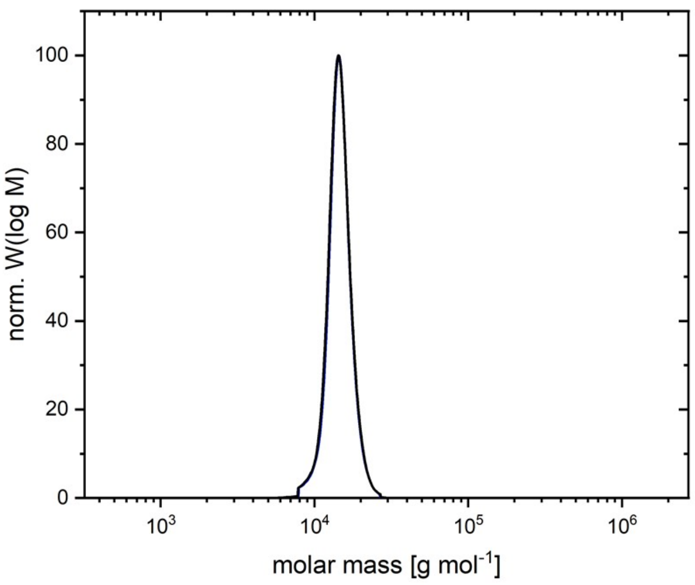

Figure S3.1.2 GPC chromatogramme of **PLLA-EAA** (measured in duplicate)

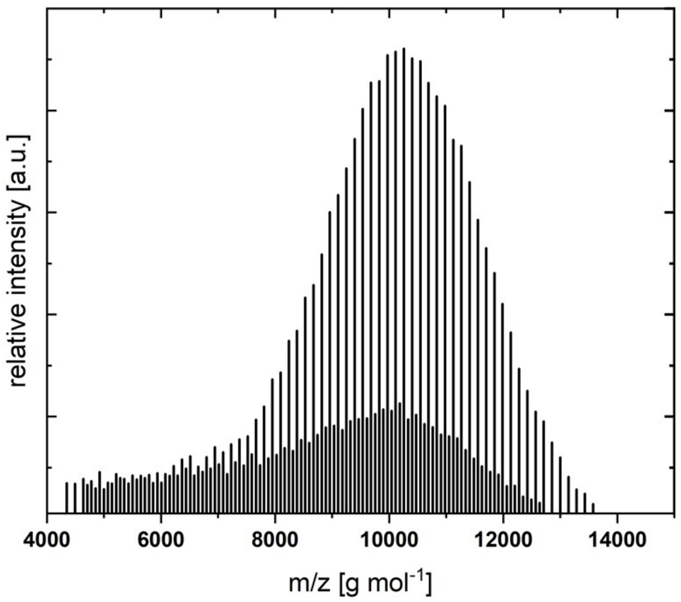

Figure S3.2.1 MALDI spectrum of **PLLA-DEAA**

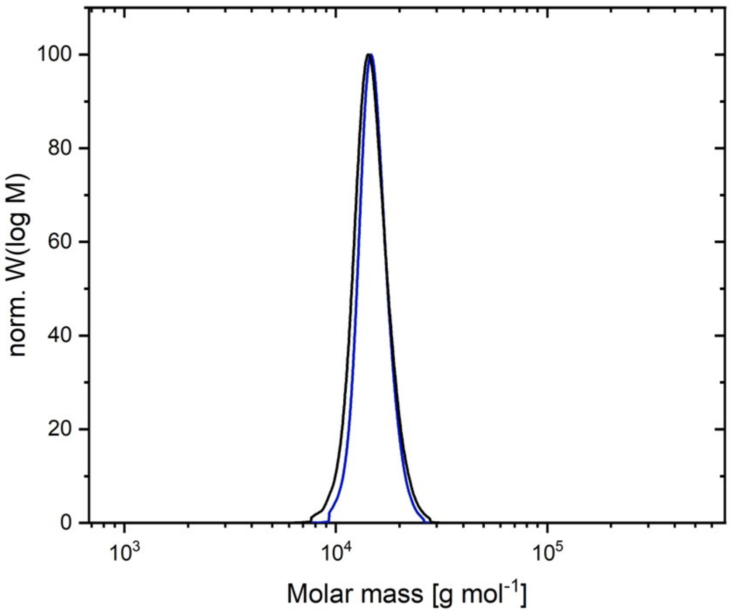

Figure S3.2.2 GPC chromatogramme of **PLLA-DEAA** (measured in duplicate)

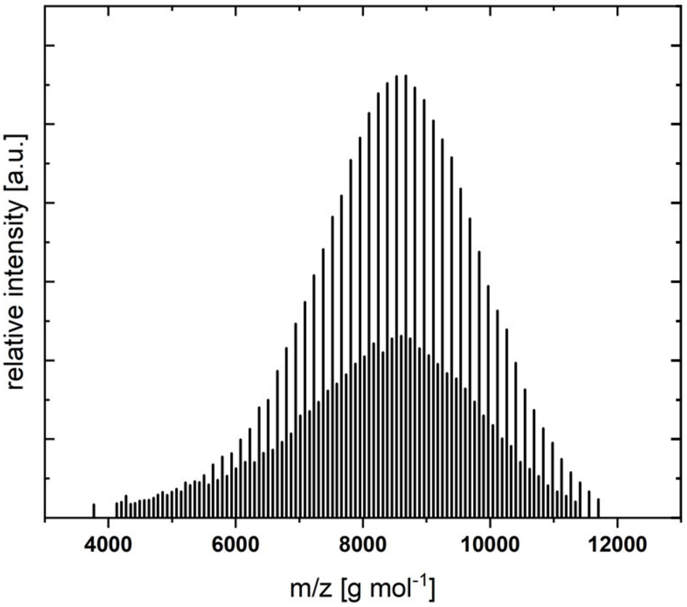

Figure S3.3.1 MALDI spectrum of **PLLA-DMAA**

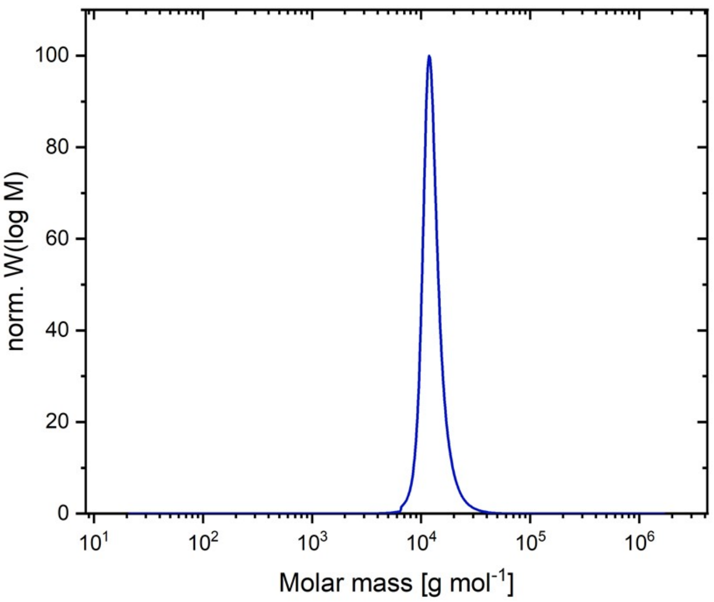

Figure S3.3.2 GPC chromatogramme of **PLLA-DMAA** (measured in duplicate)

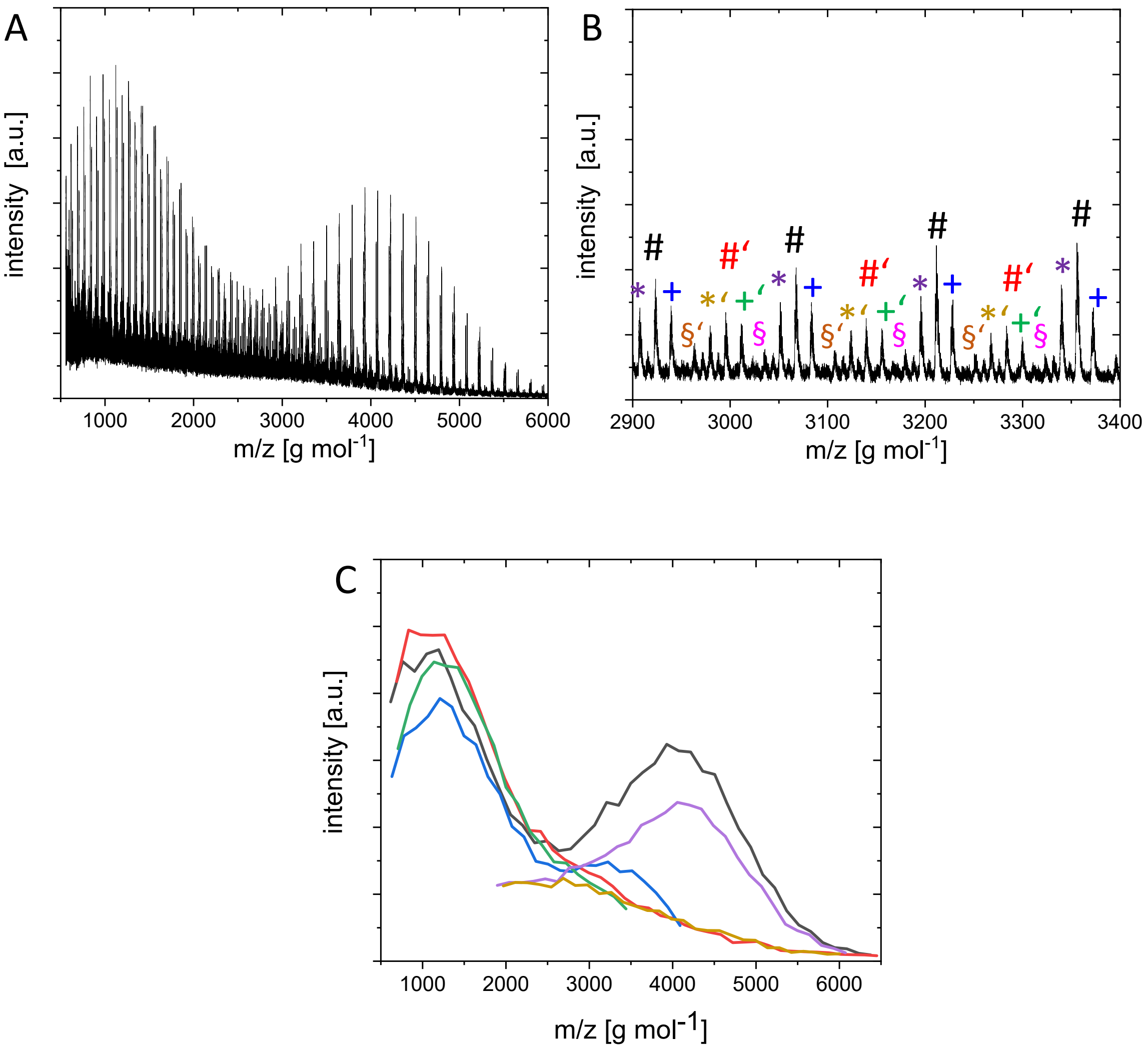

*: In-$(LA)_x$+Na^+ (x = 2n) #:[In-$(LA)_x$+K^+]*f + [H_2O-$(LA)_{x+2}$+Na^+](x = 2n) +: [H_2O-$(LA)_{x+2}$+K^+]*f (x = 2n) §: In-$(LA)_x$+H^+ (x = 2n) *‘: In-$(LA)_x$+Na^+ (x = 2n+1) #‘:[In-$(LA)_x$+K^+]*f + [H_2O-$(LA)_{x+2}$+Na^+](x = 2n+1) +‘:[H_2O-$(LA)_{x+2}$+K^+]*f (x = 2n+1) §‘ In-$(LA)_x$+H^+ (x = 2n+1)

NB: these MALDI measurements were performed on a different machine without an added Na^+ source, therefore the spectra are more complex

Figure S3.4 MALDI spectrum of **PLLA-tBuAA**

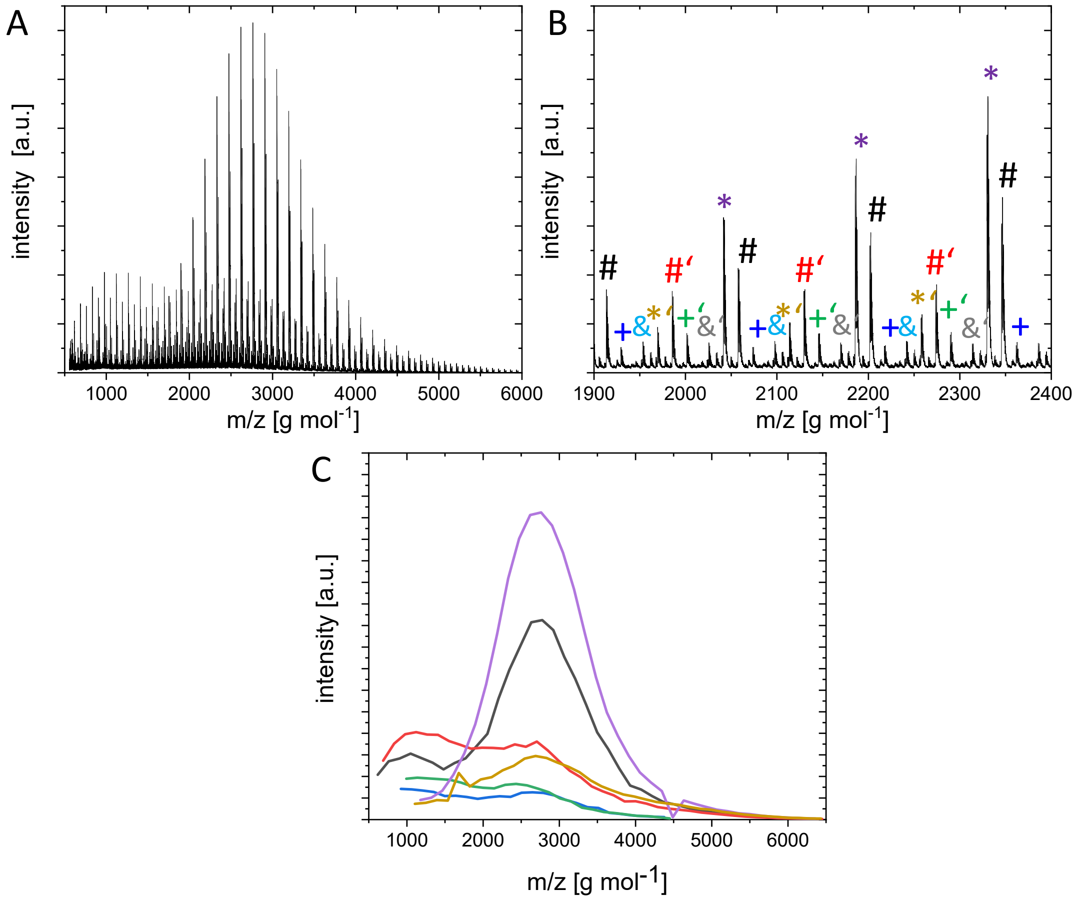

*: In-(LA)$_x$+Na$^+$ (x = 2n) #:[In-(LA)$_x$+K$^+$]*f + [H$_2$O-(LA)$_{x+2}$+Na$^+$](x = 2n) +: [H$_2$O-(LA)$_{x+2}$+K$^+$]*f (x = 2n) §: In-(LA)$_x$+H$^+$ (x = 2n) *‘: In-(LA)$_x$+Na$^+$ (x = 2n+1) #‘:[In-(LA)$_x$+K$^+$]*f + [H$_2$O-(LA)$_{x+2}$+Na$^+$](x = 2n+1) +‘:[H$_2$O-(LA)$_{x+2}$+K$^+$]*f (x = 2n+1) §‘ In-(LA)$_x$+H$^+$ (x = 2n+1), &: In-(LA)$_x$+FeOAc$^+$ (x = 2n), &’: In-(LA)$_x$+FeOAc$^+$ (x = 2n+1)

Figure S3.5 MALDI spectrum of **PLLA-AcAn**

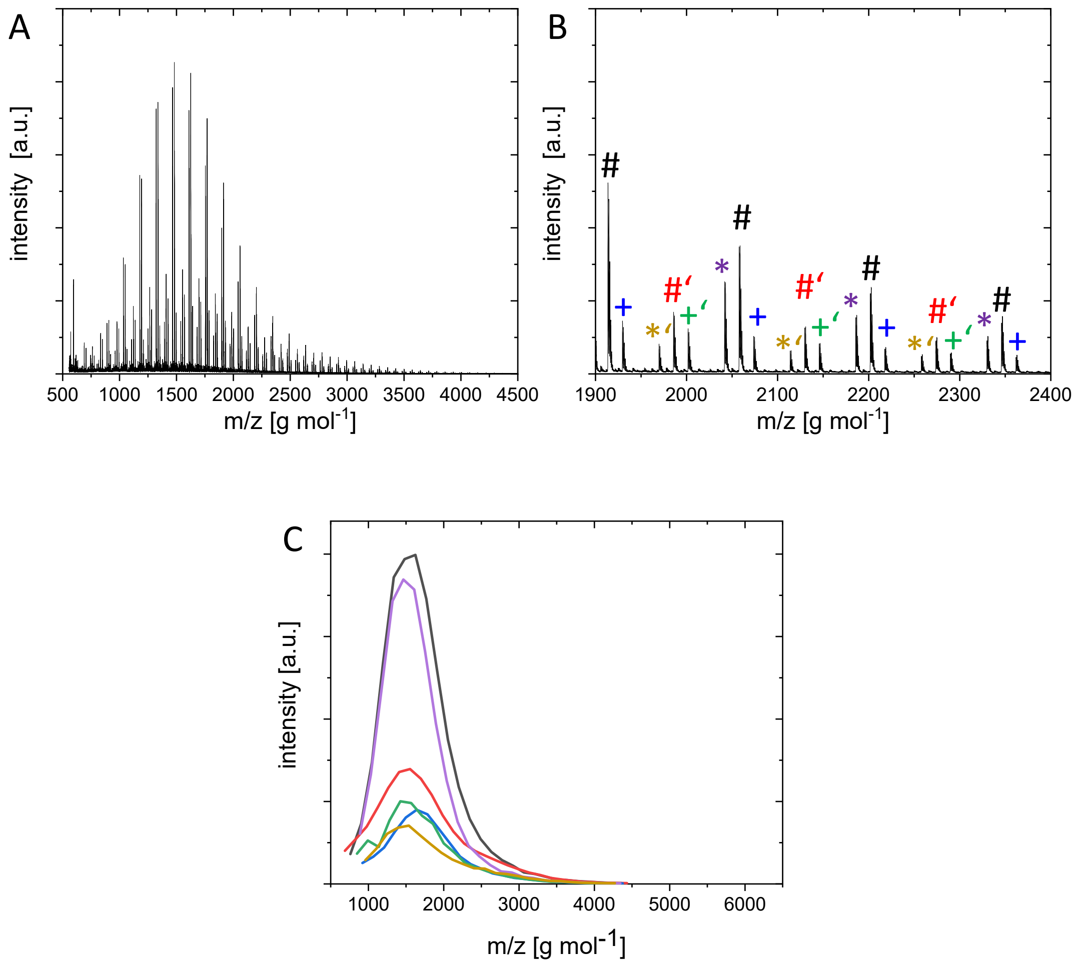

*: In-(LA)$_x$+Na$^+$ (x = 2n) #:[In-(LA)$_x$+K$^+$]*f + [H$_2$O-(LA)$_{x+2}$+Na$^+$](x = 2n) +: [H$_2$O-(LA)$_{x+2}$+K$^+$]*f (x = 2n) §: In-(LA)$_x$+H$^+$ (x = 2n) *‘: In-(LA)$_x$+Na$^+$ (x = 2n+1) #‘:[In-(LA)$_x$+K$^+$]*f + [H$_2$O-(LA)$_{x+2}$+Na$^+$](x = 2n+1) +‘:[H$_2$O-(LA)$_{x+2}$+K$^+$]*f (x = 2n+1) §‘ In-(LA)$_x$+H$^+$ (x = 2n+1).

Figure S3.6 MALDI spectrum of **PLLA-DMAAP**

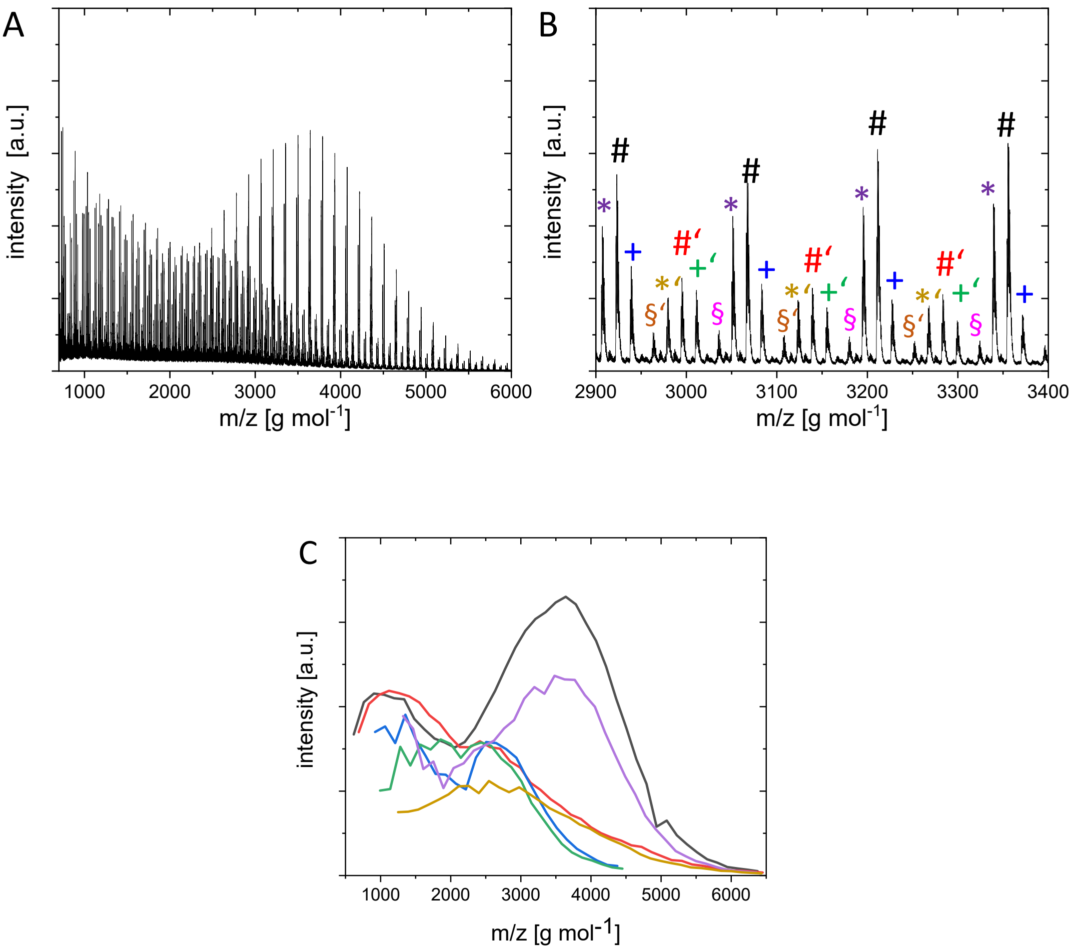

*: In-$(LA)_x$+Na^+ (x = 2n) #:[In-$(LA)_x$+K^+]*f + [H_2O-$(LA)_{x+2}$+Na^+](x = 2n) +: [H_2O-$(LA)_{x+2}$+K^+]*f (x = 2n) §: In-$(LA)_x$+H^+ (x = 2n) *‘: In-$(LA)_x$+Na^+ (x = 2n+1) #‘:[In-$(LA)_x$+K^+]*f + [H_2O-$(LA)_{x+2}$+Na^+](x = 2n+1) +‘:[H_2O-$(LA)_{x+2}$+K^+]*f (x = 2n+1) §‘ In-$(LA)_x$+H^+ (x = 2n+1).

Figure S3.7 MALDI spectrum of **PLLA-CIDEAA**

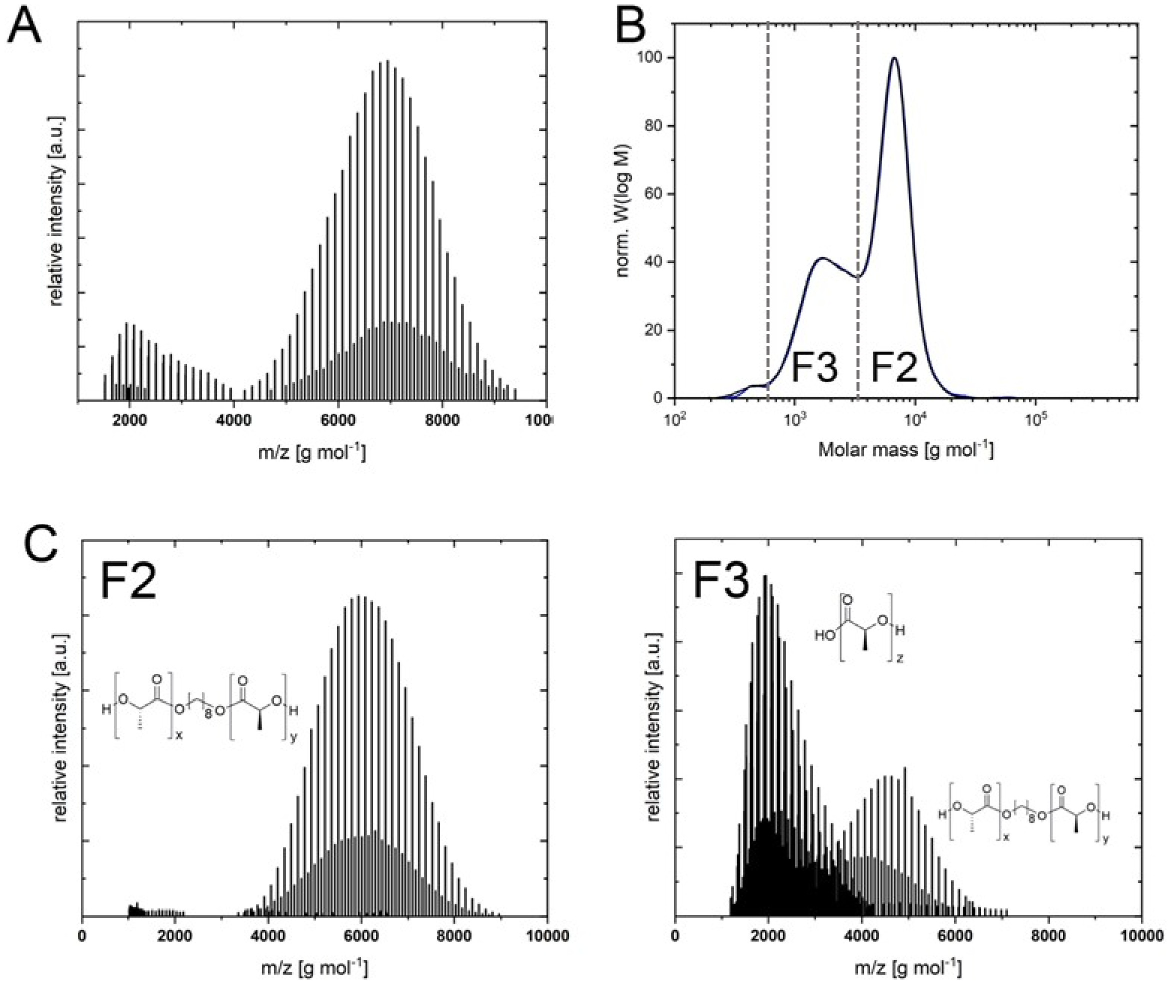

Figure S3.8 GPC and MALDI analysis of **PLLA-urea**. A) MALDI spectrum, B) GPC chromatogramme indicating the fractions collected. C) MALDI spectra of fractionations with the corresponding polymers structures.

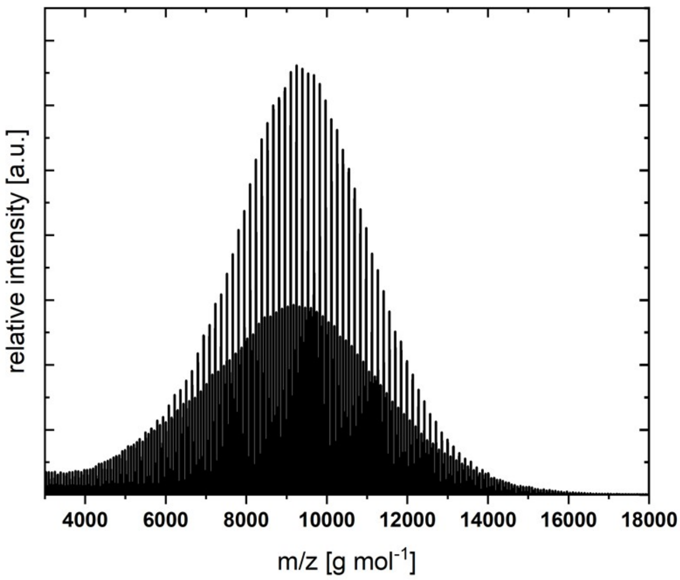

Figure S3.9.1 MALDI spectrum of **PLLA-TMU**

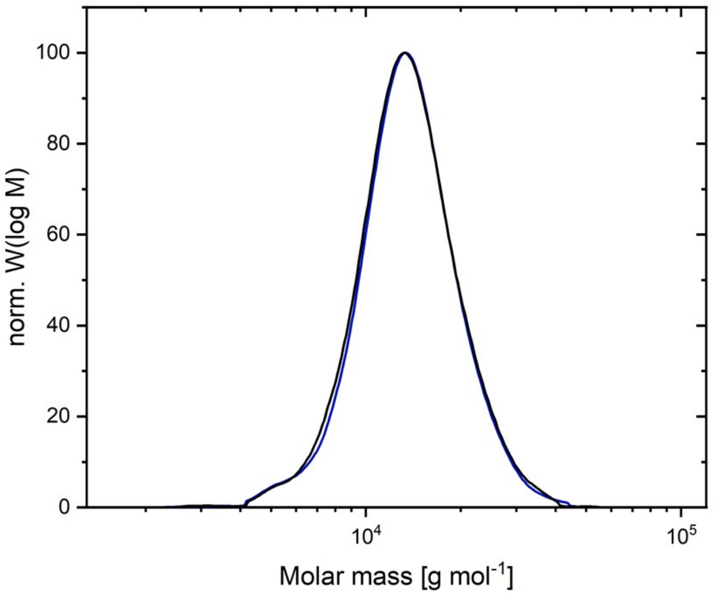

Figure S3.9.2 GPC chromatogramme of **PLLA-TMU** (measured in duplicate)

S4. Polymerisation of L-lactide with $Fe(OPv)_2$ and amides as catalyst

Table S4.1 NMR and MALDI analysis of PLLA synthesised with $Fe(OPiv)_2$

Polymer	Conversion [%]	Ini [%]	M_n NMR [g mol^{-1}]	M_n MALDI [g mol^{-1}]	M_w MALDI [g mol^{-1}]	*Đ*	2n:2n+1 [%]
**Piv-EAA**	98	84	7550	1175	1260	1.07	64
**Piv-DEAA**	89	93	10190			1.89	42
				1435	2715		
**Piv-tBuAA**	50	83	5100	2220	3180	1.43	54
**Piv-AcAn**	43	92	4330	2545	3325	1.31	53
**Piv-DMAAp**	20	86	1800		Not acquired		
**Piv-ClDEAA**	32	46	2440	2635	3005	1.14	66
**Piv-Urea**	86	40	2400		Not aquired		
**Piv-DMUrea**	73	23	1040		Not acquired		

S5. MALDI spectra and GPC chromatogrammes of PLLA with targeted molecular weights

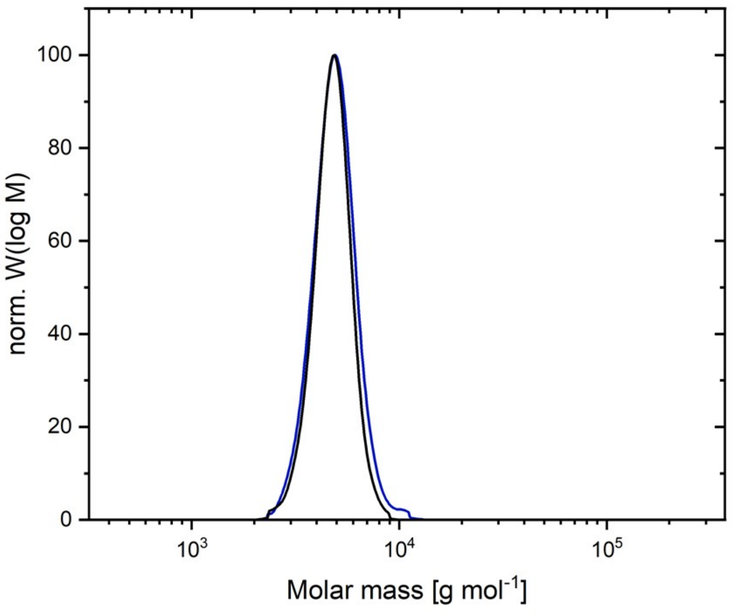

Figure S5.1.1 GPC chromatogramme of **PLLA-DEAA-5k** (measured in duplicate)

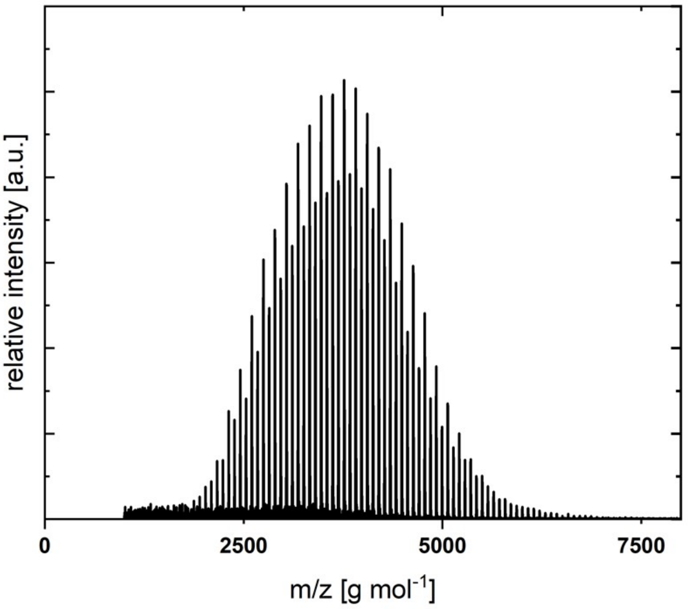

Figure S5.1.2 MALDI spectrum for **PLLA-DEAA-5k**

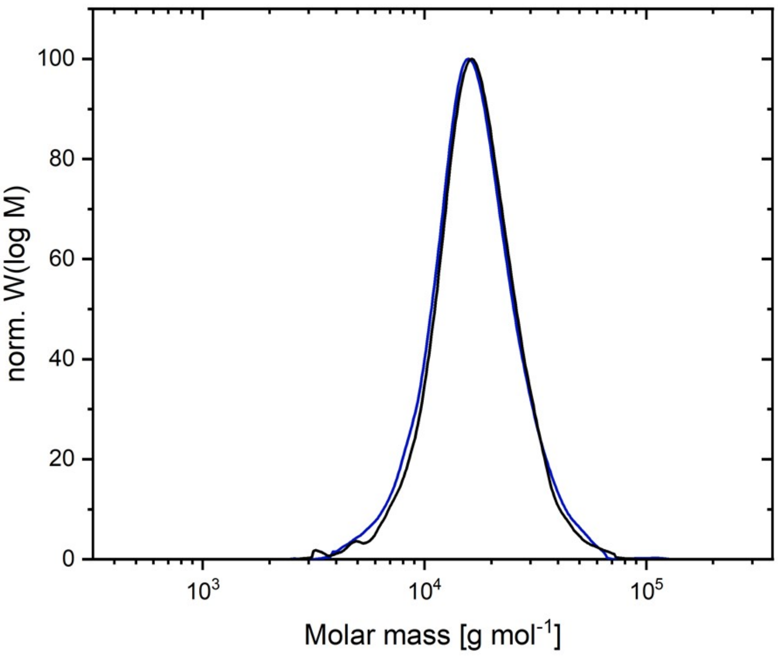

Figure S5.2.1 GPC chromatogramme of **PLLA-DEAA-15k** (measured in duplicate)

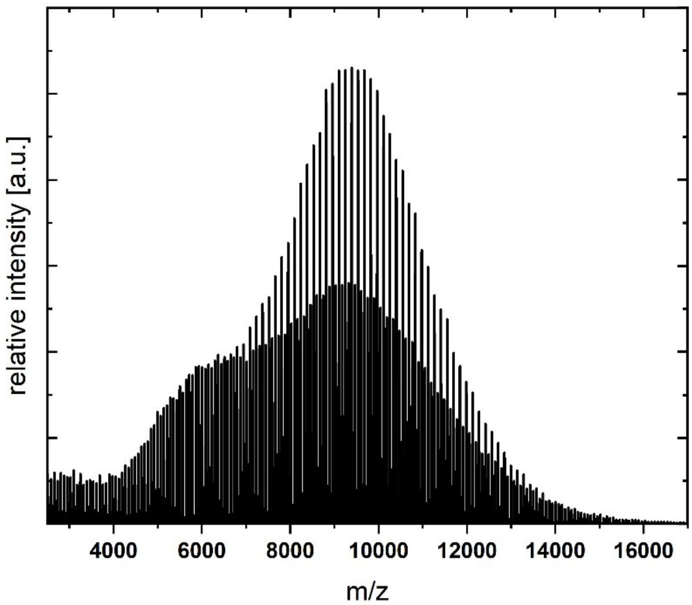

Figure S5.2.2 MALDI spectrum for **PLLA-DEAA-15k**

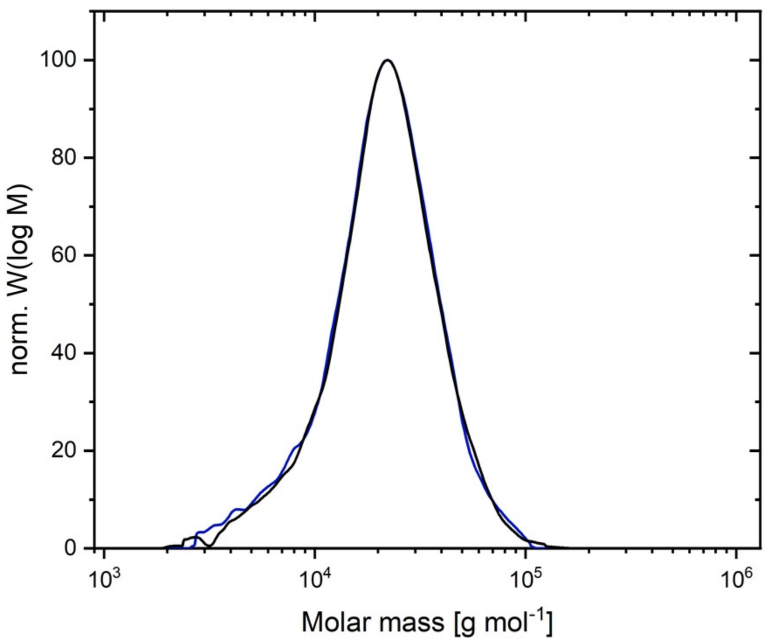

Figure S5.3.1 GPC chromatogramme of **PLLA-DEAA-25k** (measured in duplicate)

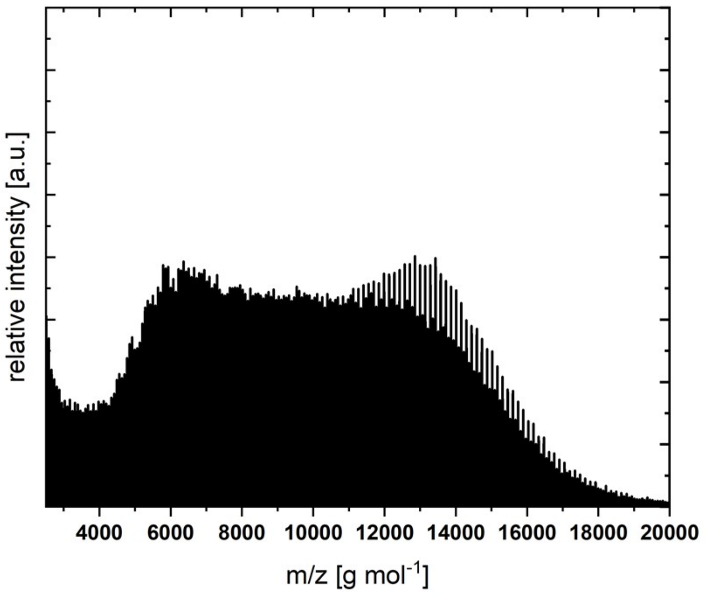

Figure S5.3.2 MALDI spectrum for **PLLA-DEAA-25k**

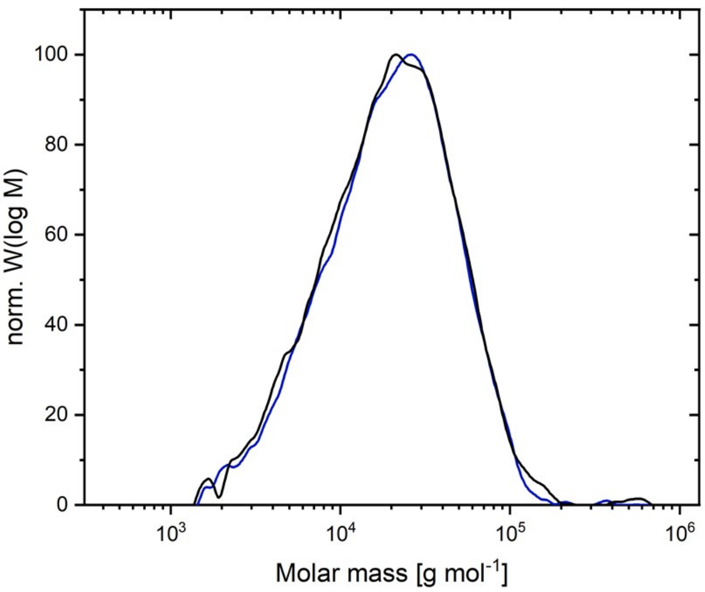

Figure S5.4.1 GPC chromatogramme of **PLLA-DEAA-50k** (measured in duplicate)

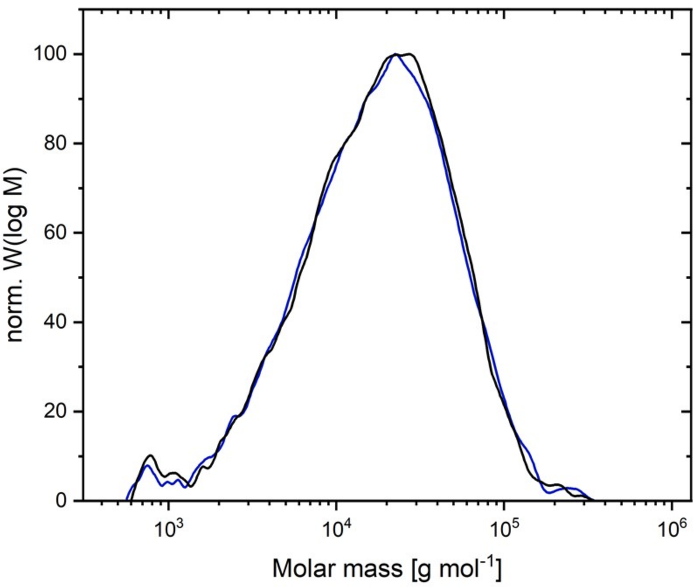

Figure S5.5.1 GPC chromatogramme of **PLLA-DEAA-100k** (measured in duplicate)

S6. Kinetics of Polymerisations of lactide (L-LA$_2$) and caprolactone (CL) by the catalyst system.

Homopolymerisations were performed with DEAA as amide at 105 °C for (L-LA)$_2$ and 105 °C and 140 °C for CL. Additionally the rates were determined per monomer for a 1:1 copolymerisation mixture. The conversion of monomers was calculated by integration of the ^{1}H-NMR spectrum. For lactide the methine Hα peak was used (q at 5.16 - 5.13 ppm for polymer plus terminal Hα (4.35 – 4.31 ppm) against 5.05 - 5.03 ppm for monomer) and for CL the ε-CH$_2$ peak (4.07-4.03 ppm) against the monomer at 4.23-4.20 ppm)

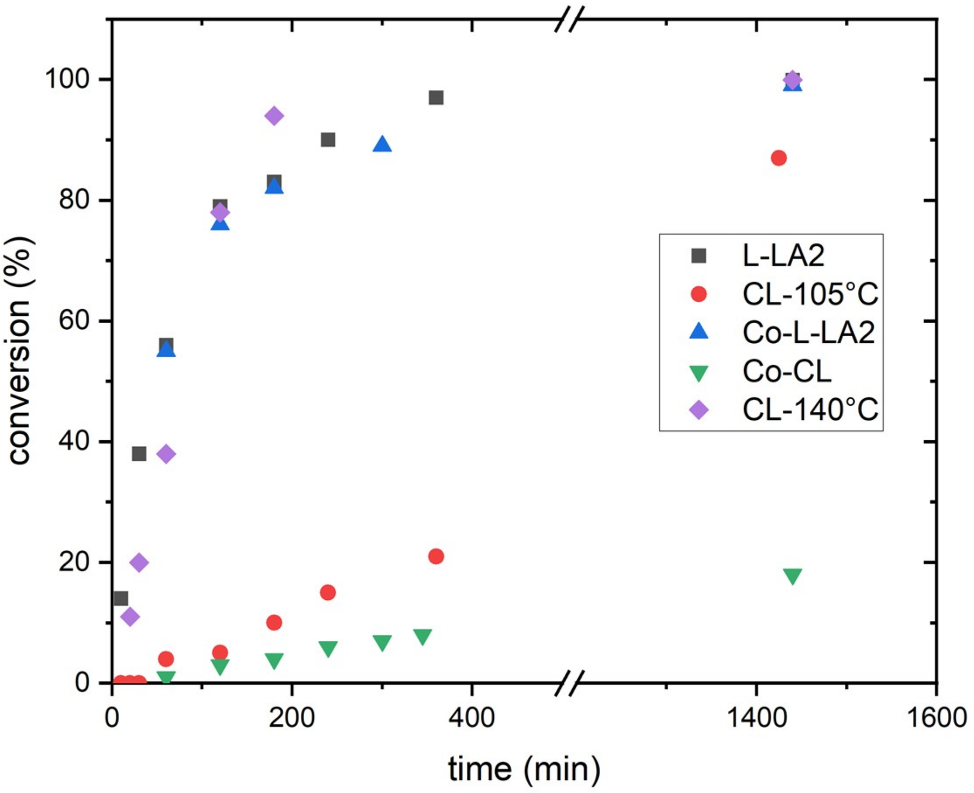

S7. NMR spectrum of copolymer indicating the peaks used for calculation of molar ratios, randomness and average chain lengths

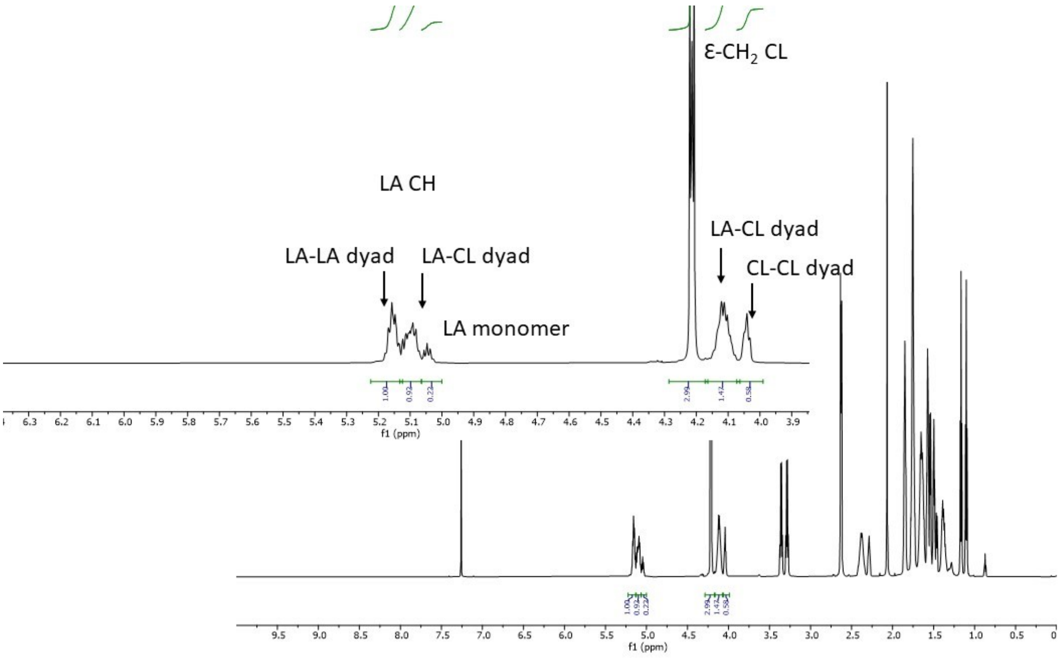

S8. GPC chromatogrammes of homo- and copolymers

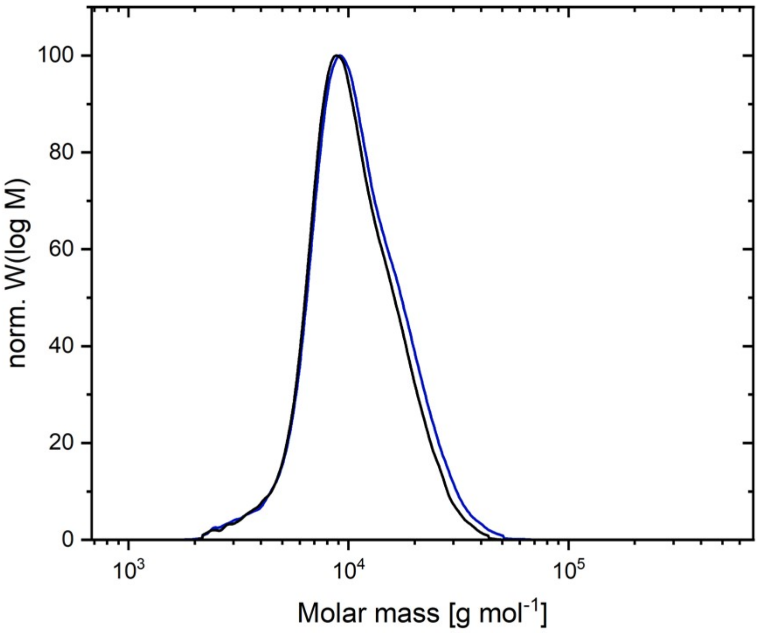

Figure S8.1 GPC chromatogramme of **PLLA-DEAA-hex** (measured in duplicate)

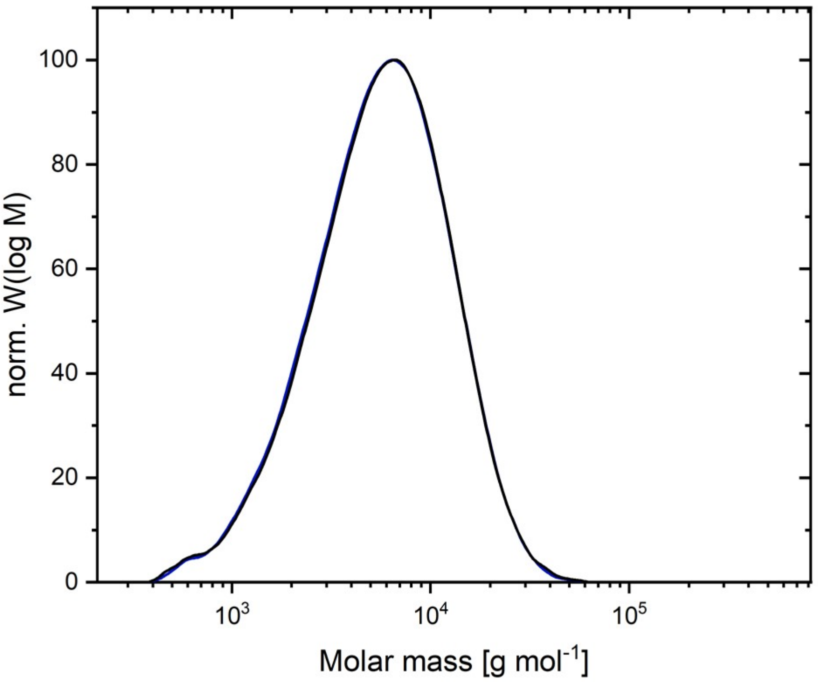

Figure S8.2 GPC chromatogramme of **PCL-DEAA-hex** (measured in duplicate)

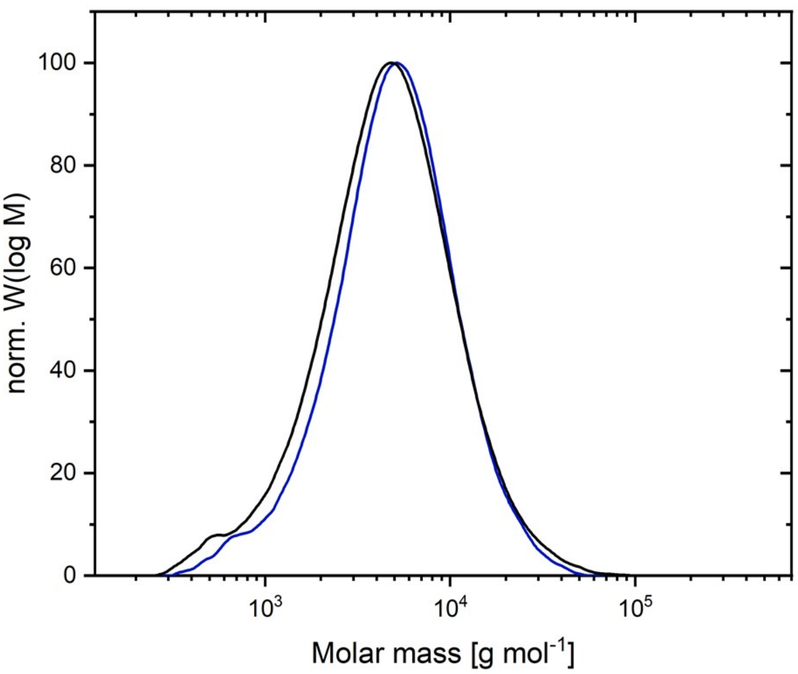

Figure S8.3 GPC chromatogramme of **PLLA-*grad*-PCL-105** (measured in duplicate)

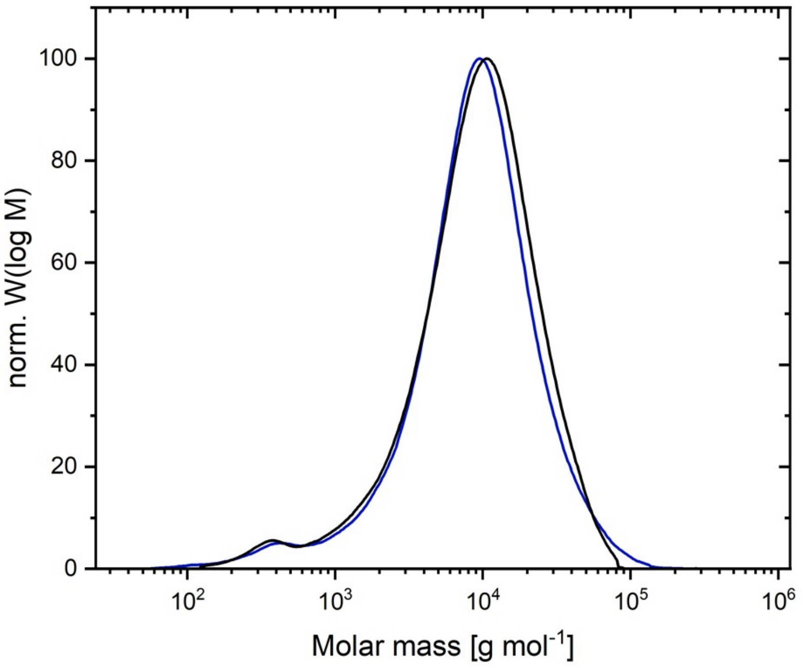

Figure S8.4 GPC chromatogramme of **PLLA-*grad*-PCL-140** (measured in duplicate)

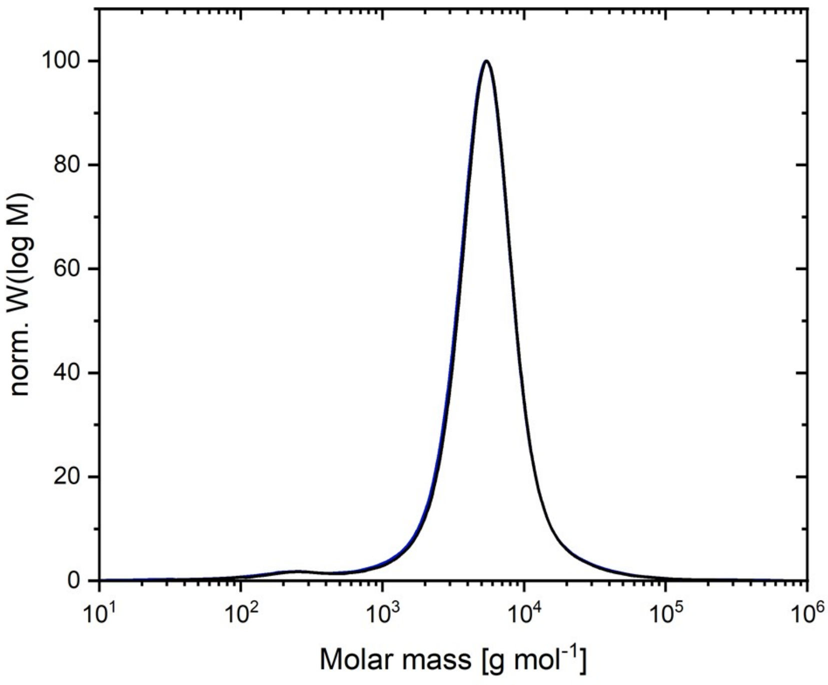

Figure S8.5 GPC chromatogramme of **PLLA-*b*-PCL-105** (measured in duplicate)

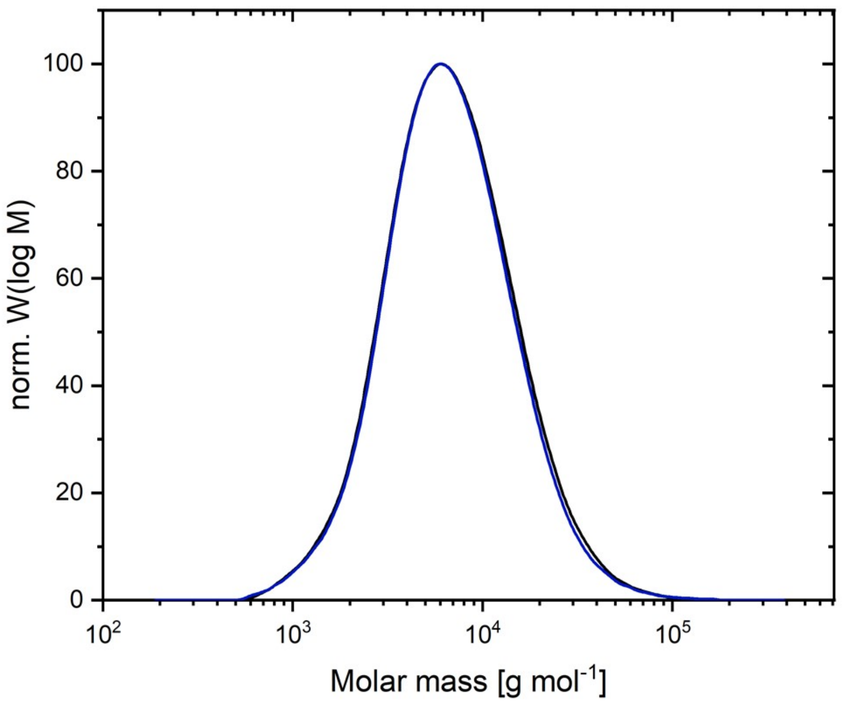

Figure S8.6 GPC chromatogramme of **PCL-*b*-PLLA-140** (measured in duplicate)

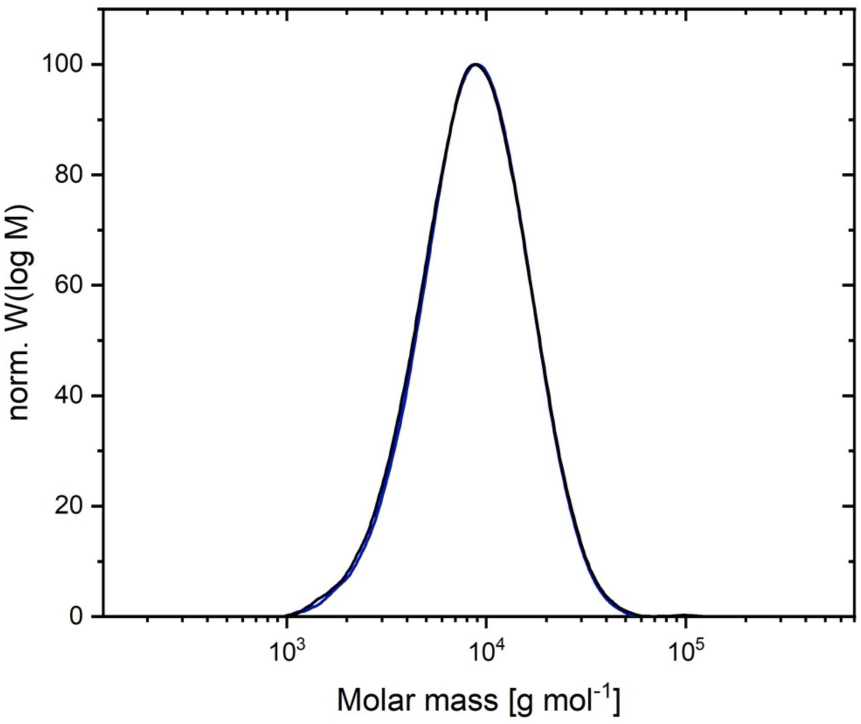

Figure S8.7 GPC chromatogramme of **PCL-*b*-PLLA-140-105** (measured in duplicate)

S9. Selected MALDI spectra of copolymers

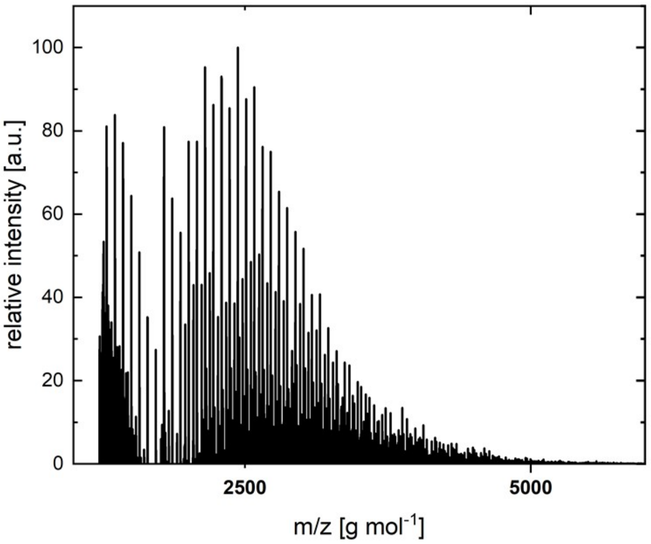

Figure S9.1 MALDI spectrum of **PLLA-*grad*-PCL**

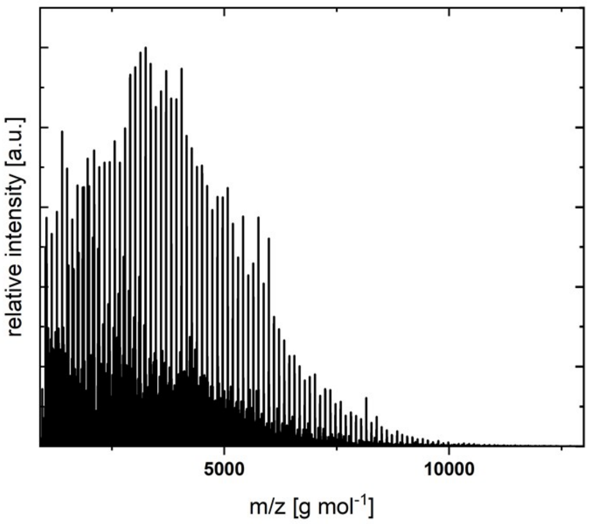

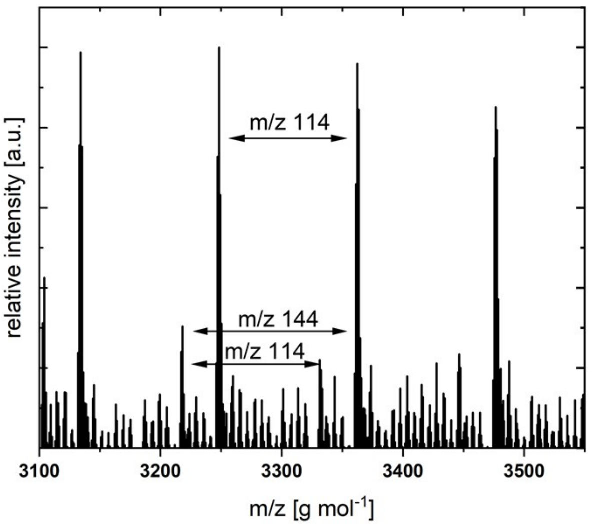

Figure S9.2 MALDI spectrum and excerpt of **PCL-*b*-PLLA-140-105**

S10. Glycolide copolymerisation

Table S10.1 PLGA copolymerisation data

Type	Reaction time	Temp [°C]	Monomer Feed (LA:GL)	Conv LA [%]	Conv GL [%]	Molar ratio LA:GL	(LA-GL) rel. molar fraction	I_{LA}	I_{GL}	R
PLLGA	25 h	105	1:1	98	>99	56:44	0.174	6.4	5.1	0.35

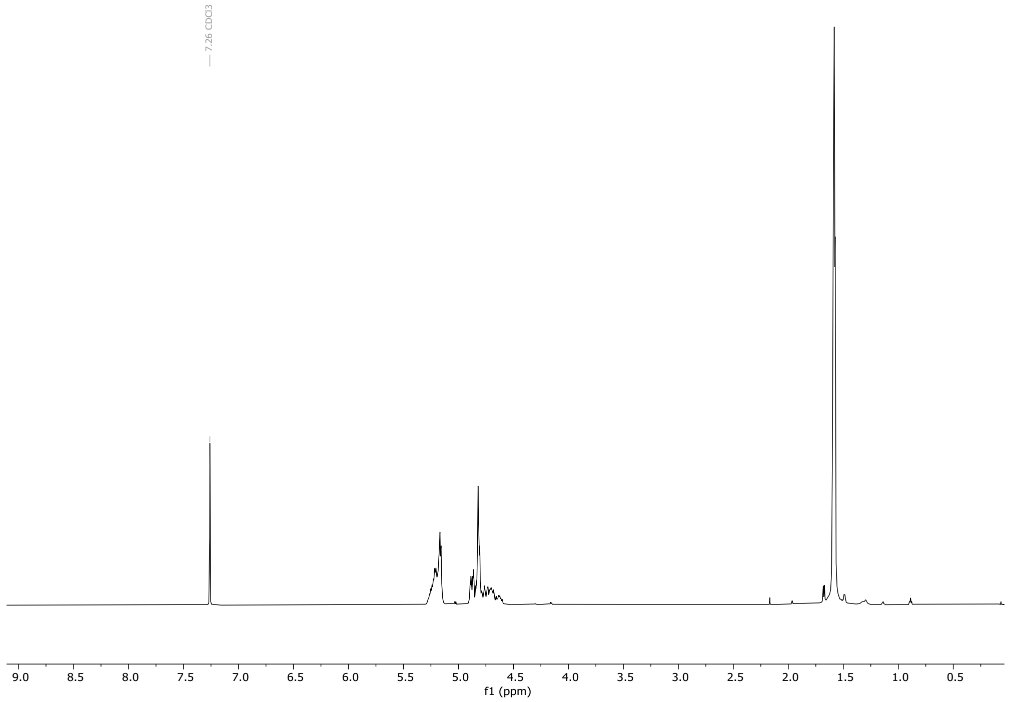

Figure S10.1 ^{1}H-NMR spectrum of **PLLGA** ($CDCl_3$)

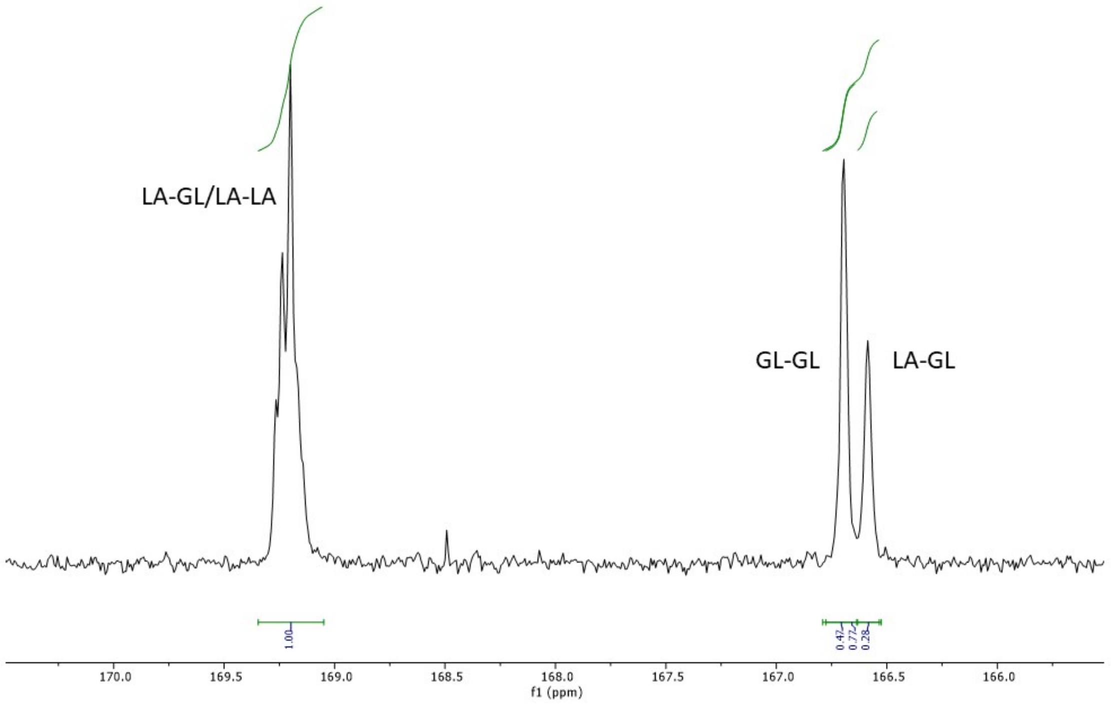

Figure S10.2 ^{13}C-NMR spectrum excerpt (in DMSO-d_6)

S11. DSC investigations

Table S11.1 Thermal data of representative polymers.

Polymer	Tm_1 [°C]	ΔH_{m1} [Jg^{-1}]	Tm_2 [°C]	ΔH_{m2} [Jg^{-1}]	ΔH_c [Jg^{-1}]	X_{PLLA}	X_{PCL}
**PLLA**	163-168	59	-	-	-	63%	-
**PLLA-*b*-PCL**	33	3	-	-	2	-	2%
**PCL-*b*-PLLA-140-105**	51	45	142	10	-	20%	37%
**PLLA-*grad*-PCL-105**	37	10	113	2	9	4%	2%

Data are taken from the second heating and temperatures are peak values.

Crystallinity X was calculated using

$$X = \frac{\Delta Hm - \Delta Hc}{\omega\, \Delta Hm^0} x\, 100$$

where ΔH_m and ΔH_c are the melting and cold crystallisation enthalpies taken from the DSC, ΔH_m^0 is the melting enthalpy for a 100% crystalline polymer (93.7 Jg^{-1} for PLLA[1] and 135 Jg^{-1} for PCL[2]) and ω is the weight fraction of the respective polymer.

References

[1] S. Jia, D. Yu, Y. Zhu, Z. Wang, L. Chen, L. Fu, Morphology, Crystallization and Thermal Behaviors of PLA-Based Composites: Wonderful Effects of Hybrid GO/PEG via Dynamic Impregnating, Polymers (Basel) 9(10) (2017).

[2] T.M. Diez-Rodriguez, E. Blazquez-Blazquez, N.L.C. Antunes, M.R. Ribeiro, E. Perez, M.L. Cerrada, Nanocomposites of PCL and SBA-15 Particles Prepared by Extrusion: Structural Characteristics, Confinement of PCL Chains within SBA-15 Nanometric Channels and Mechanical Behavior, Polymers (Basel) 14(1) (2021).
